# Pangenomic insights into Dehalobacter evolution and acquisition of functional genes for bioremediation

**DOI:** 10.1099/mgen.0.001324

**Published:** 2024-11-20

**Authors:** Olivia Bulka, Radhakrishnan Mahadevan, Elizabeth A. Edwards

**Affiliations:** 1 Department of Chemical Engineering and Applied Chemistry, University of Toronto, Toronto, Ontario, Canada

**Keywords:** bioremediation, *Dehalobacter*, pangenome, reductive dehalogenase

## Abstract

*Dehalobacter* is a genus of organohalide-respiring bacteria that is recognized for its fastidious growth using reductive dehalogenases (RDases). In the SC05 culture, however, a *Dehalobacter* population also mineralizes dichloromethane (DCM) produced by chloroform dechlorination using the *mec* cassette, just downstream of its active RDase. A closed genome of this DCM-mineralizing lineage has previously evaded assembly. Here, we present the genomes of two novel *Dehalobacter* strains, each of which was assembled from the metagenome of a distinct subculture from SC05. A pangenomic analysis of the *Dehalobacter* genus, including RDase synteny and phylogenomics, reveals at least five species of *Dehalobacter* based on average nucleotide identity, RDase and core gene synteny, as well as differential functional genes. An integration hotspot is also pinpointed in the *Dehalobacter* genome, in which many recombinase islands have accumulated. This nested recombinase island encodes the active RDase and *mec* cassette in both SC05 *Dehalobacter* genomes, indicating the transfer of key functional genes between species of *Dehalobacter*. Horizontal gene transfer between these two novel *Dehalobacter* strains has implications for the evolutionary history within the SC05 subcultures and of the *Dehalobacter* genus as a whole, especially regarding adaptation to anthropogenic chemicals.

**Impact Statement**

*Dehalobacter restrictus* is a key microbe in many microbial communities for bioremediation. The first *Dehalobacter* strain was isolated over 25 years ago, but increased accessibility of genomics has rapidly improved the discovery of additional strains in this genus. Here, we show that these many strains belong to distinct evolutionary lineages and propose reclassification into multiple species. We find a relationship between lineage and key functional enzymes for dechlorination and find evidence of the transfer of genetic cargo between species with shared activity. Overall, this research provides an initial insight into the evolution of the *Dehalobacter* genus and has implications for the use of genomics in applied contexts.

## Data Summary

The authors confirm that all supporting data, code and protocols have been provided within the article or through supplementary data files. All reads and assemblies are available on the National Center for Biotechnology Information under BioProject PRJNA1013980. These *Dehalobacter* genomes are deposited under accession numbers CP148031 (strain DAD) and CP148032 (strain SAD). This Whole Genome Shotgun project is deposited in GenBank under the accession no. JBAWSR000000000 (SC05-UT) and JBAWSS000000000 (DCME). Illumina paired reads are deposited in the Sequence Read Archive under accession numbers SRR27458442 (SC05-UT) and SRR27458441 (DCME).

## Introduction

*Dehalobacter* is a genus of organohalide-respiring bacteria (OHRB) in the Bacillota phylum (formerly Firmicutes) and a common dechlorinator of chlorinated organics for bioremediation. Initial characterization revealed that these reductive dechlorinators each harness a reductive dehalogenase (RDase) to dechlorinate a specific chlorinated compound, using hydrogen or formate as an electron donor [1,4]. This restricted metabolism earned this microbe the species name *restrictus*, which is the only taxonomically characterized and validated species within the genus to date [1]. While many strains of *Dehalobacter* have no designated species name, many are referred to interchangeably as *Dehalobacter restrictus*, both formally and casually.

As of December 2023, at least 36 strains of *Dehalobacter* have been experimentally or genomically characterized (Table S1, available in the online version of this article), and many more have been detected through amplicon sequencing in environmental surveys and enrichment cultures. Of these, 25 genomes in various stages of completion are publicly available in the National Center for Biotechnology Information (NCBI) or Integrated Microbial Genomes & Metagenomes (IMG/M) databases (Table S1), many of which are metagenome-assembled genomes (MAGs). These genomes are ~3 Mb, with a GC content of ~44%, and typically contain three to five rRNA operons. *Dehalobacter* strains have been isolated from contaminated and pristine groundwater and sediment sites around the world, from China to the Netherlands to Australia to Canada and the USA, indicating their early proliferation as a genus despite relying on electron acceptors generally seen as anthropogenic. For example, the type strain, *D. restrictus* PER-K23, subsists solely on perchloroethene (PCE) as an electron acceptor – a chlorinated ethene with no known natural origin whose production only began in the 1940s [1,2]. Adaptation to new electron acceptors may occur through horizontal gene transfer (HGT) or duplication and diversification of RDases.

RDases use their catalytic subunit (RdhA) to reductively remove a halogen group (archetypally chlorine) from an organohalide substrate. These enzymes are ubiquitous in OHRB, and each *Dehalobacter* genome encodes 10–40 *rdhA* genes, though high expression is usually limited to one RDase when growing on a specific substrate [5,8]. Each RDase has a different substrate preference. The genus’s breadth of electron acceptors ranges from relatively simple chlorinated alkanes and alkenes [like chloroform (CF), trichloroethane (TCA), dichloroethane (DCA) and trichloroethene] to chlorinated aromatics like chlorinated benzenes and β-hexachlorocyclohexane (β-HCH) [5,7, 9,12]. RdhA sequences are classified into orthologue groups (OGs), wherein each OG comprises all RdhA sequences within 90% aa identity that have additional support from phylogenetic clustering [13]. A database has been developed to host this classification (rdasedb.biozone.utoronto.ca) [14]. Such classification revealed RDase synteny within the genomes of another OHRB – *Dehalococcoides mccartyi –* and unveiled the relationships between several RDase OGs, including substrate adaptation, duplication within genomes and HGT from other bacterial lineages [14].

HGT is a main driver for evolution in Bacillota, which tend to express conjugation machinery and have a large ‘mobilome’ compared to other phyla [15,19]. Mobile genetic elements (MGEs) carrying a number of gene cassettes for biodegradation have been found [20,23], and the transfer of PceA between *Dehalococcoides* strains [21,24] firmly establishes HGT as critical for the evolution of OHRB for bioremediation. Though MGEs can be identified in *Dehalobacter* genomes, evidence of their transfer has not been directly shown within this genus. The most prevalent MGEs in *Dehalobacter* genomes are transposases, which are proposed mediators of dechlorination gene transfer. One RDase operon encoding *pceABCT* was detected between inverted repeats in strains CF and DCA and between two cryptic transposases in strain PER-K23, indicating a potential ancient HGT event [25,26]. Only a handful of *Dehalobacter* genomes encode the *mec* cassette – a series of genes involved in dichloromethane (DCM) dechlorination [27] – but transposases are also interspersed in this gene neighbourhood, implying HGT of this cassette despite its apparent lack of function in most of the *Dehalobacter* strains that harbour it [28].

Three studies have linked DCM degradation in a microbial community to *Dehalobacter* growth [29,31]. Upon further genomic characterization, one of these DCM-degrading ‘*Dehalobacter*’ strains was distinguished as a new genus distinct from *Dehalobacter: Candidatus* Dichloromethanomonas elyunquensis [32]. This reclassification reinstates the need for genomic information or isolation for complete certainty in the taxonomy of a 16S sequence fragment and casts doubt on the taxonomy of the other two reported DCM-degrading *Dehalobacter* strains. To date, neither of these strains have been genomically characterized or isolated. One was lost from its mixed culture completely when enriched further on DCM and was replaced by a confirmed DCM degrader in another novel genus: *Candidatus* Formimonas warabiya [33]. The third report described *Dehalobacter* in two related enrichment cultures, SC05-UT and DCME [28,29].

SC05-UT reduces CF to DCM, which is further mineralized to carbon dioxide and hydrogen. This hydrogen provides the electron donor to support further CF reduction through ‘self-feeding’ [29,34]. DCME mineralizes DCM as a sole substrate, but in both cultures, *Dehalobacter* was identified as the sole dechlorinator [29]. The metagenome of each subculture was previously assembled, and one *Dehalobacter* MAG emerged from each [28]. One contig in each MAG encodes the only expressed RDase in the culture (named *acdA* in SC05-UT; dechlorinates CF to DCM) as well as the *mec* cassette (involved in DCM mineralization), but no closed genomes arose from the assembly [28]. Without a complete genome, neither biotransformation could be attributed to a single strain. Multiple closely related strains of *Dehalobacter* could be working in tandem despite the detection of only a single *Dehalobacter* 16S sequence when degrading either CF or DCM as a sole substrate [29]. In this work, we set out to close and characterize (what we expected to be) the genome of a single *Dehalobacter* strain performing both biotransformation steps in SC05 and orient this organism in relation to the *Dehalobacter* pangenome.

## Methods

### SC05 subculture establishment

SC05 is a commercially used anaerobic mixed-microbial enrichment culture (also known as KB-1^®^ Plus CF) originally sampled from a site polluted with chlorinated ethenes and ethanes in California in 2010 by SiREM (Guelph, ON), after which it was enriched on CF [29,34]. An aliquot of this culture, referred to as SC05-UT, has been maintained at the University of Toronto without the addition of any electron donor since 2018 as previously described [29]. Originally transferred from SC05-UT, the DCME culture has been enriched by feeding DCM alone since 2019 [29].

### DNA extraction, sequencing and assembly

Cultures were sampled in September and November 2020 (Fig. S1) for DNA extraction. DNA samples were sequenced by the Genome Quebec Innovation Centre using both Illumina NovaSeq 6000 and PacBio Sequel II technologies (Illumina Inc., San Diego, CA) as described in our related resource announcement [35]. Additional details are provided in Text S1. These samples, herein referred to as ‘round 1’, were sequenced using a workflow that included PCR amplification, leading to uneven ‘wavy’ read coverage (example shown in Fig. S2), which impeded binning and closing *Dehalobacter* MAGs (assembly described in [35]). For this reason, additional samples were sequenced in March 2023 (‘round 2’), which is expanded upon in a subsequent resource announcement [36]. DNA from round 2 was sent to the Genome Quebec Innovation Centre for Illumina NovaSeq 6000 sequencing (Illumina Inc.). After sequencing, hybrid assemblies were constructed with hybridSPAdes [37] using PacBio long reads from round 1 [36]. Additional quality control, mapping and binning were performed using Anvi’o v7’s metagenome Snakemake pipeline [38,40]. Ultimately, the SC05-UT *Dehalobacter* MAG from round 1 and the DCME *Dehalobacter* MAG from round 2 were further curated as described below.

### Genomic closing and curation

Each MAG was closed through contig extension via read mapping of the PacBio HiFi long reads and by end matching using blast. Briefly, PacBio HiFi reads were mapped to each contig, using the Geneious aligner (Geneious v8.1.9, https://www.geneious.com). Overhangs were extracted, aligned with muscle [41], and the consensus sequences were appended to the end of the contig. Extended contigs were assembled using the Geneious *de novo* assembler, or by blast of each contig end against all metagenomic contigs to identify small contigs missed during binning. Contig order was quality checked using iRep to determine GC skew and through manual inspection compared to other closed *Dehalobacter* genomes using Mauve Aligner in Geneious [42,43]. Genes were annotated with The NCBI Prokaryotic Genome Annotation Pipeline v6.7 [44], and taxonomy was determined using Genome Taxonomy Database (GTDB) R220 [45,46]. Circular genomes were visualized with GenoVi [47] and were deposited in the NCBI under accession numbers CP148031 (strain DAD) and CP148032 (strain SAD).

### Pangenomics and phylogenomics

This *Dehalobacter* pangenomic analysis included the two new genomes described above, as well as all unique publicly available *Dehalobacter* genomes and assemblies in the NCBI GenBank and IMG databases surpassing a quality threshold (Text S2). This threshold was set to include all circularized genomes (strains CF, DCA, 12DCA and PER-K23), or genomes in multiple contigs with low contamination (<10%) and high completion (>95%) (Table S2). The *Dehalobacter* pangenome was compiled and analysed using the Anvi’o pangenomics workflow to detect and cluster core genes in all high-quality *Dehalobacter* genomes [38]. Homologous gene clusters were detected in the pangenome, and a subset of 42 single-copy core genes with high geometric homogeneity (i.e. gap/residue distribution within a gene cluster independent of aa identity) and low functional homogeneity (i.e. conservation of aligned aa residues across genes) were selected and used to estimate the relationships between *Dehalobacter* strains through phylogenomic analysis. A maximum-likelihood (ML) phylogenetic tree was constructed with IQ-TREE, using the Whelan and Goldman substitution model and 1000 bootstraps [48]. The average nucleotide identity (ANI) was calculated between all genome pairs in both directions using pyANI to reduce the impact of reference bias and algorithmic variability [49]. Annotated 16S rRNA genes were extracted from each *Dehalobacter* genome and aligned using muscle v3.8.31 [41]. An ML tree was created using RAxML, with the gamma model of rate heterogeneity and 100 rapid bootstrap replicates followed by ML optimization [50].

### RDase classification and phylogenetic comparisons

All *rdhA* genes were extracted from each selected *Dehalobacter* genome, translated and confirmed to contain the RDase domain defined in Pfam as PF13486, as well as at least one iron–sulphur cluster motif (CXXCXXXC). Pseudogenes, truncated genes and sequences less than 200 aas were excluded from further analysis, producing 407 total RDases across genomes. Each of these RdhA sequences was assigned to an OG using a 90% aa identity cutoff after BLASTing against an in-house RDase database (http://rdasedb.biozone.utoronto.ca). Ambiguous OGs were resolved by inclusion in specific clades using a phylogenetic tree, in which these sequences were aligned with muscle v3.8.31 before a maximum likelihood tree was built with RAxML with the gamma model of rate heterogeneity and 100 rapid bootstrap replicates followed by ML optimization [41,50]. An RDase from *Nitratireductor pacificus* was used as an outgroup (WP_008597722.1). The best scoring tree was used as the final tree. This RDase tree was further analysed using the Anvi’o interactive viewer to confirm or amend the OG assignment [38]. Specific OGs were further analysed using GeneRax [51], a maximum-likelihood species-tree aware phylogenetic inference software that accounts for sequence-level and gene-level events like HGT. Species-aware gene trees were visualized with Thirdkind [52], after which ‘back-in-time’ transfers were manually removed. In circularized genomes and in long contigs of fragmented genome assemblies, adjacent RdhA sequences (within 10 kb of each other) were also concatenated and aligned to visualize OG synteny across *Dehalobacter* genomes. The concatenated sequences were aligned and visualized using clinker [53].

### Detection of mobile and conjugative elements

Conjugative elements were detected using ConjScan and TXSSScan [54,56]. Phages were predicted using PHASTER [57]. The insertion sites of interest were detected by finding the closest core gene on either end of the *mec* cassette and extracting these regions in each genome, as described in reference [54]. These genomic islands were aligned and further annotated using proMGE [58].

### PCR and quantitative PCR detection of MGE and strain variation

Primers were designed to amplify key genome regions to detect the integration and mobilization of the mobile element in each *Dehalobacter* genome (Table S3, Fig. S10). PCR was performed on metagenomic DNA from SC05-UT and DCME with each primer set using 2× Taq MasterMix (FroggaBio, Vaughan, ON) according to the manufacturer’s specifications and visualized via agarose gel electrophoresis.

To distinguish closely related strains, primers were also designed for a core gene in *Dehalobacter* with high sequence variability: flagellar basal body rod protein, *flgC* (Table S3). Using these primers, quantitative PCR (qPCR) was performed on each metagenomic DNA sample and archived DNA samples dating back to the cultures’ establishments. The qPCR reaction mixtures were prepared in a UV-treated PCR cabinet (ESCO Technologies, Hatboro, PA) and contained 10 µl of 2× SsoFast EvaGreen® (Bio-Rad Laboratories, Hercules, CA), forward and reverse *flgC* primers (0.25 µM each) and 2 µl of template DNA. The amplification programme and analyses were conducted using a CFX96 Touch Real-Time PCR Detection System and the CFX Manager software (Bio-Rad Laboratories). The qPCR method included an initial denaturation step at 98 °C for 2 min, followed by 40 cycles of 5 s at 98 °C and 10 s at 55 °C, including a 2 °C/s ramp between temperatures. The quantification was performed using tenfold serial dilutions of PCR-produced standard DNA amplified from a larger region of the *flgC* gene. The number of copies was calculated assuming a 100% DNA extraction efficiency.

## Results and discussion

### Distinct *Dehalobacter* genomes assembled from each subculture

**Fig. 1.**
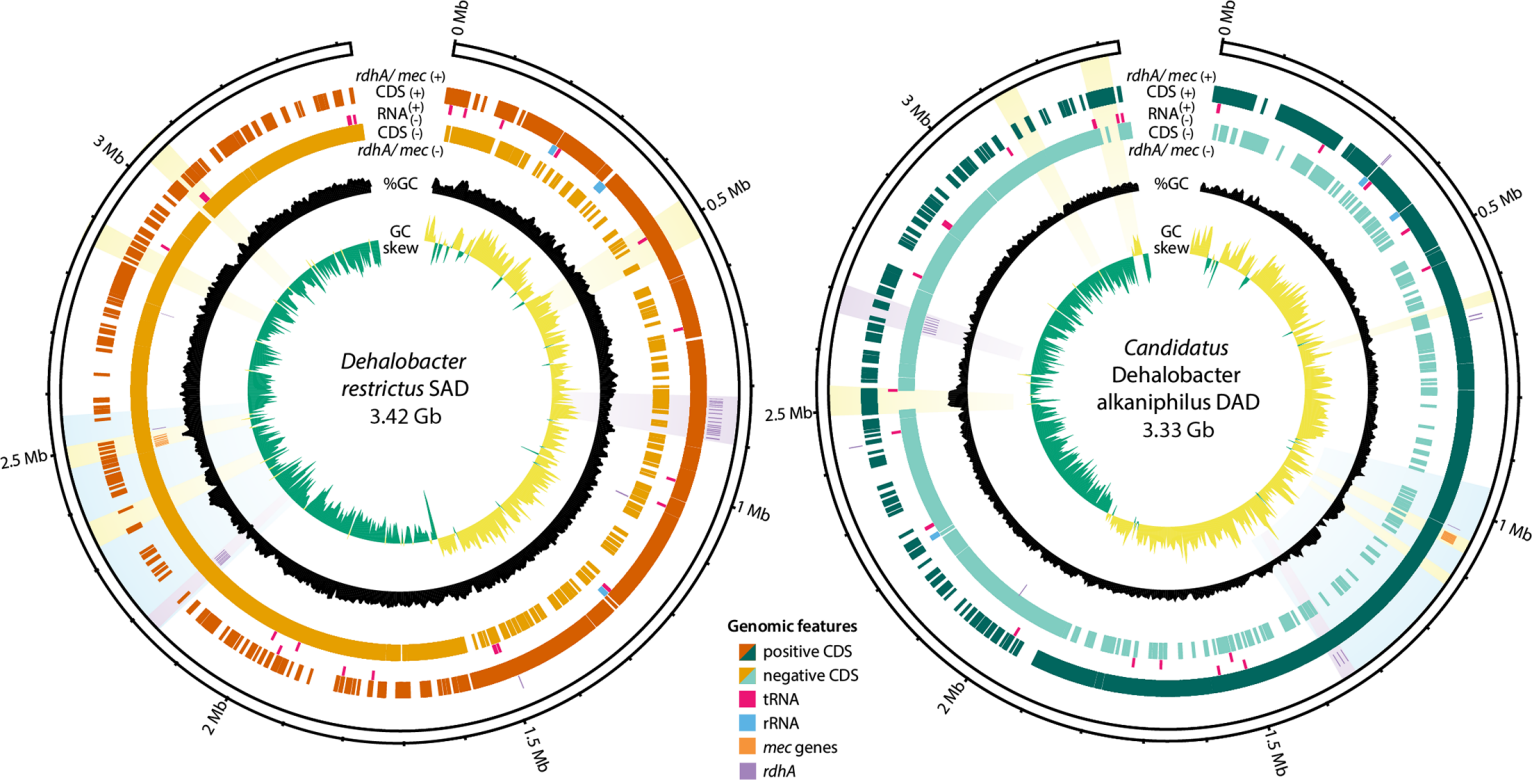
Genome map of *Dehalobacter* strains SAD and DAD. From inside outwards: (1) GC skew; (2) %GC; – strand: (3) select coding sequences (CDSs), (4) all CDS and (5) RNA genes; + strand: (6) RNA genes, (7) all CDS and (8) select CDS. Select CDS include *mec* genes (orange) and RDases (purple). Highlighted regions include predicted prophages (yellow), RDase-dense regions (purple) and a predicted genomic island (blue).

Two unique *Dehalobacter* genomes were assembled: one from the SC05-UT culture metagenome and one from the DCME culture metagenome (Fig. 1). The **S**C05-UT–**a**ssembled ***D****ehalobacter* genome, *D. restrictus* strain SAD, is 3.42 Mb long, with a GC content of 44.2% and 3394 predicted genes. The **D**CME–**a**ssembled ***D****ehalobacter* genome, *Dehalobacter* sp. strain DAD, is 3.33 Mb long and 44.9% GC with 3311 predicted genes. The genome of strain SAD encodes 4 complete rRNA operons and 56 tRNA genes, compared to 3 rRNA operons and 52 tRNA genes in strain DAD. Strain SAD also encodes 27 RDases, while strain DAD only contains 17. Between strains, no RDase is identical, and only six pairs of RDases are >90% identical (i.e. belonging to the same OG). Strain SAD encodes two RDases belonging to OG150, and strain DAD encodes two OG96 RDases; no other OGs have multiple RDases within one genome. Previous work characterized the active RDase in strain SAD – named AcdA from OG97 – which dechlorinates CF to DCM; strain DAD expresses an AcdA homologue with five aa differences dubbed ‘BcdA’ [28]. Both of these genomes also encode a complete *mec* cassette for DCM dechlorination, consistent with previous findings in the draft assemblies [28].

Despite originally deriving from the same parent culture, these genomes are quite divergent, with several large genome rearrangements. Paired reads from each subculture metagenome do not map to the genome assembled from the other subculture, which also demonstrates that each subculture metagenome only contains one *Dehalobacter* strain. These strains have an ANI of only 95.3% – barely crossing the typically accepted threshold for a single species [59]. This implies an early divergence of these lineages rather than more recent evolution in the lab enrichment culture.

### Speciation in the *Dehalobacter* pangenome: one becomes five

The dearth of *Dehalobacter* genomic data has precluded pangenomic analysis of the genus. Prior to this work, only four closed genomes were publicly available – two of which (strains CF and DCA) are extremely similar (99.8% ANI) due to evolutionarily recent strain divergence events [25]. Here, the addition of two new closed genomes and supplementation with publicly available high-quality multi-contig MAGs allow an exploration of genus diversity.

**Fig. 2.**
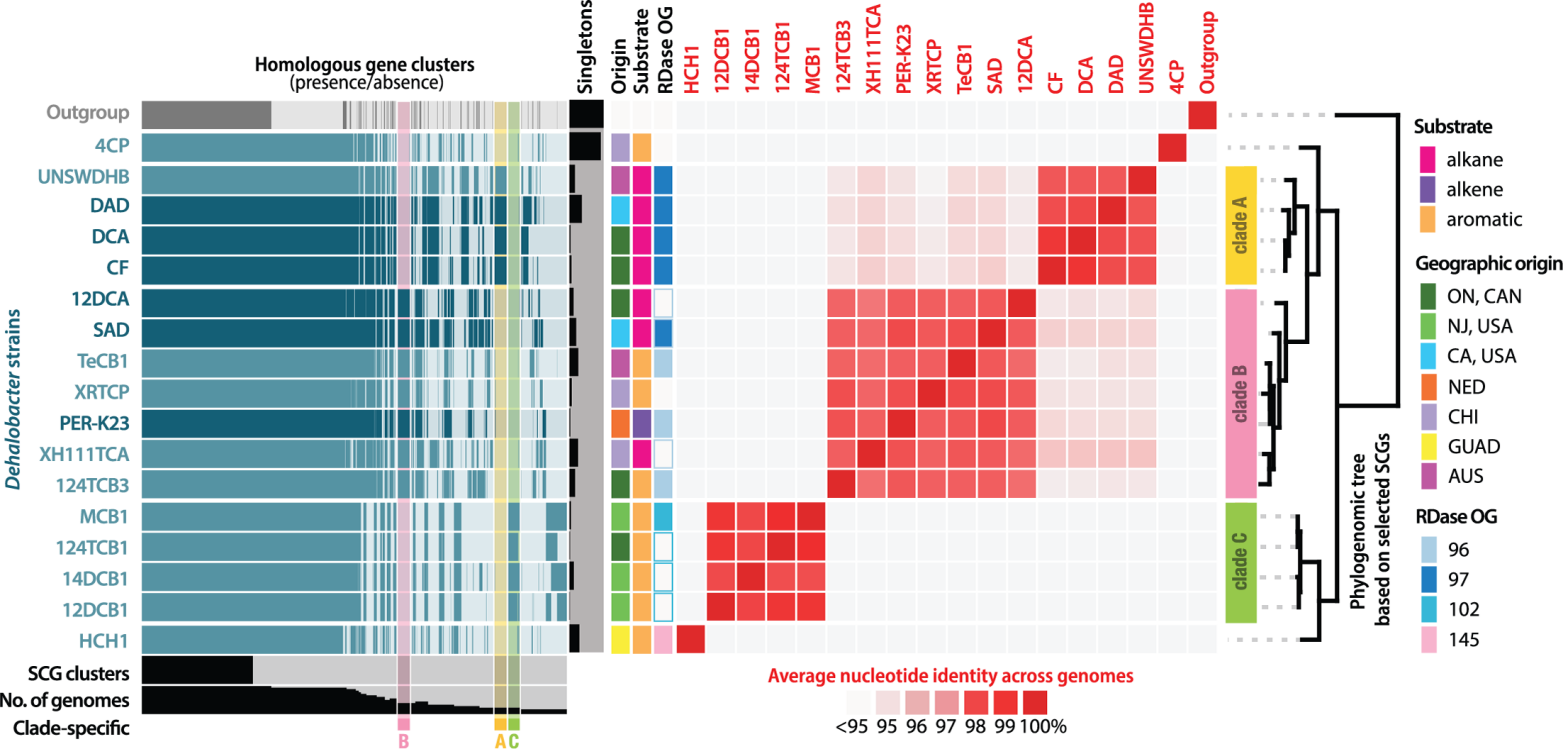
Pangenomic analysis of the *Dehalobacter* genus. Homologous gene clusters are shown in blue, with single-copy core genes (SCGs) and clade-specific genes highlighted in their respective colours. Closed genomes are dark blue, while multi-contig genomes are light blue. The outgroup (*Ca*. D. elyunquensis: LNDB000000000) is shown in grey. ANI is in red, and multi-strain clades of the phylogenomic tree are highlighted on the right. The active RDase OG in each strain is shown with a shaded box; an open box represents the gene presence of an OG expected to be active but lacking characterization data. Geographic origins are abbreviated as follows: CAN, Canada; NED, Netherlands; CHI, China; GUAD, Guadeloupe; and AUS, Australia.

Seventeen high-quality *Dehalobacter* genomes with diverse substrates and geographic origins were compiled to create the pangenome, resulting in three distinct clades comprising more than one genome (Fig. 2). Two genomes evaded clustering into these distinct groups – strains 4CP and HCH1 – suggesting evolutionary distance between these two strains and any other genomically characterized *Dehalobacter*. Clade A comprises strains UNSWDHB, DCA, CF and DAD, each of which is well described experimentally. The genomes of all clade A strains except strain UNSWDHB are circularized. Clade B, which includes the type strain *D. restrictus* PER-K23, also comprises strains 12DCA, TeCB1, XRTCP, XH111TCA, 124TCB3 and SAD, of which strains 12DCA, PER-K23 and SAD have circularized genomes. Clade C has no representative complete genome, consisting of strains MCB1, 124TCB1, 12DCB1 and 14DCB1.

The ANI was calculated between all strains (Fig. 2). Within each clade, all genomes shared high ANI (clade A: >98.7%, clade B: >98.0% and clade C: >99.1%), while the ANI was much lower across clades (91–96%). The ANI has long been used as a proxy for speciation, with 95% as a typical species cutoff [59]. Using this cutoff as a ‘species’ definition, four to five species of *Dehalobacter* may be represented in this pangenome. Five species are also supported by the GTDB [45, 46], wherein different species placeholder names have been assigned to clades A, B and C, as well as strain 4CP (Table S2). Strain HCH1 was not included in the GTDB, as it is not deposited on the NCBI. Though all *Dehalobacter* strains have historically been referred to – informally, if not formally as well – under only one species name, *restrictus*, here, we propose five species of *Dehalobacter*, grouped as follows. Strains HCH1 and 4CP are each distinct species from their nearest relative, and clades A, B and C are each a distinct species, where clade B refers to the *restrictus* type. Here, we propose the names ‘*Candidatus* Dehalobacter alkaniphilus’ sp. nov. and ‘*Candidatus* Dehalobacter aromaticus’ sp. nov. to describe clades A and C, respectively, reflecting their preferential electron acceptors (Fig. 2).

A phylogenetic tree of 16S rRNA genes also supports the distinction of clades A and B, despite their closer relation than the other clades. Their 16S sequences share only 92–93% identity between clades (Fig. S3). The topology of this 16S-based tree differs slightly from the protein gene tree presented in Fig. 2, where strain 4CP branches more closely to *D. restrictus* (clade B) than the clade A sequences. Clade-specific gene clusters were also extracted and compared in Text S3. Clade A genomes encoded more metabolic genes – including aspartate ammonia lyase, fumarate reductase and a nitrogen fixation cassette – and clade B encompassed a larger mobilome (Fig. S5). Overall, phylogenetically and functionally, clades A and B are distinct lineages despite their closer phylogenomic relationship than to other *Dehalobacter* species.

**Fig. 3.**
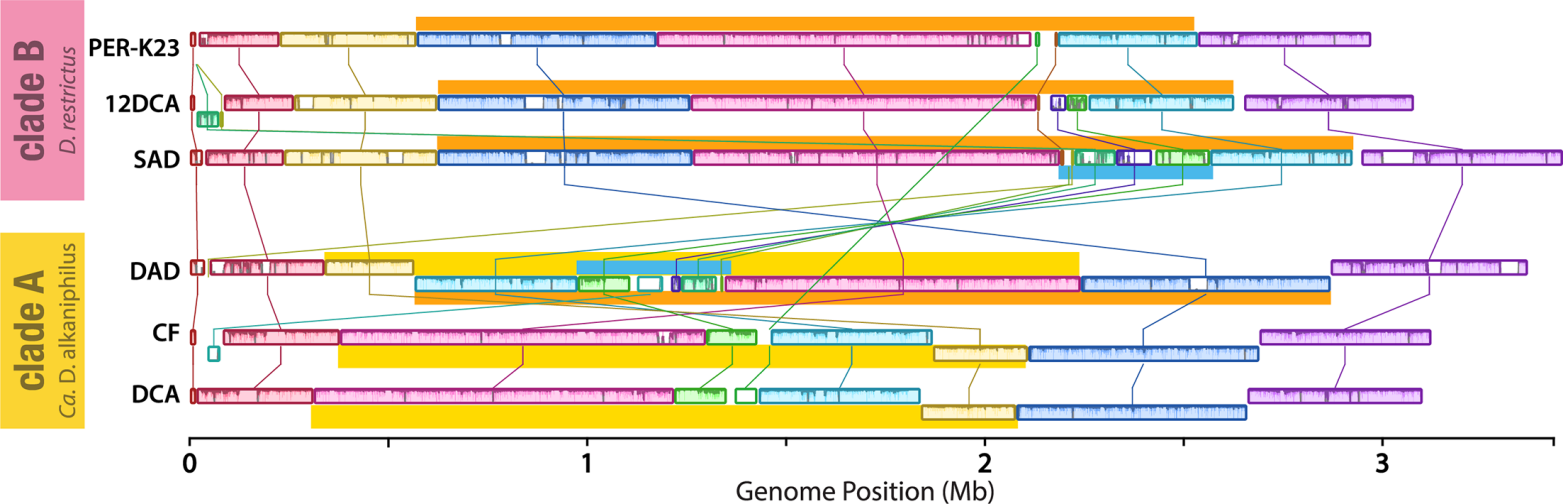
Alignment of closed *Dehalobacter* genomes representing two clades. Each coloured brick represents one LCB. Highlights in yellow and orange mark inversions between strain DAD and strains in clades A and B, respectively. Highlighted in blue is a region common only to strains SAD and DAD.

Circularized genomes from clades A and B were also aligned to visualize genome colinearity. Locally colinear blocks (LCBs) in the genomes within clade B (*D. restrictus*) are ordered consistently, as are those within clade A (*Ca*. D. alkaniphilus). There is one large-scale genome rearrangement between strain DAD and the other strains in its clade (Fig. 3, marked in yellow). Interestingly, strains CF and DCA have an incongruent GC skew in this region, which can be corrected by an inversion between two rRNA operons [25]. This suggests a relatively recent rearrangement event, wherein the LCB order of strain DAD may represent an intermediary stage between that of clade B and that of strains CF and DCA. In fact, with one additional rearrangement between two inverted insertion sequences (Fig. 3, highlighted in orange), the two clades have identical LCB order [25]. Notably, there is one unique region only common to strains SAD and DAD, compared to other known strains (Fig. 3, highlighted in blue), which is discussed in detail below. In addition to clade-specific genome rearrangements, the similarity within each LCB is higher between genomes of the same clade compared to across clades (closer inspection of example LCBs in Fig. S6), further differentiating the two clades. It is clear from the inclusion of multi-contig *Dehalobacter* MAGs in the *Dehalobacter* pangenome that the genus extends beyond these two clades; thus, additional closed genomes in clade C (and beyond) are necessary for a full view of genus-wide *Dehalobacter* synteny.

### Organization of the *Dehalobacter* RDase-ome

**Fig. 4.**
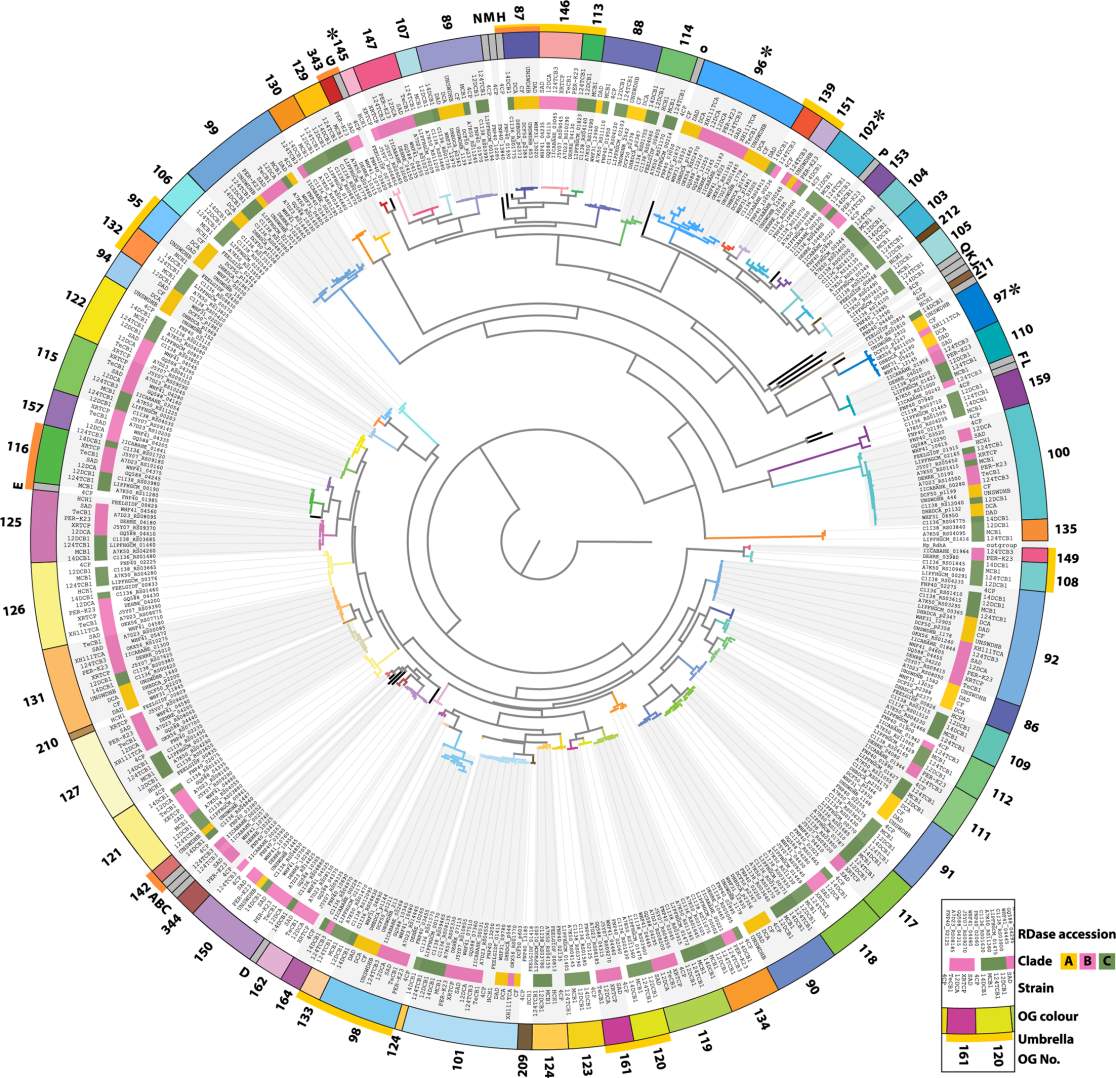
Phylogenetic tree of all *Dehalobacter* RdhA sequences from high-quality genomes. Leaves are labelled with the RdhA accession and *Dehalobacter* strain and are coloured by *Dehalobacter* clade and OG classification. Singletons are shown in grey and labelled A–Q. Stars indicate an OG including at least one characterized RDase; outer yellow highlights denote OG umbrellas; orange highlights indicate singleton umbrellas.

To explore the evolution of RDases in *Dehalobacter*, all putative *rdhA* sequences from the *Dehalobacter* pangenome were translated for phylogenetic OG categorization (Fig. 4). Two new OGs were defined: OG343 (WHF41_10730, DEHRE_10315) and OG344 (WHF41_10740, DEHRE_10325, FNP40_03410). Several pairs of OGs were overlapping (i.e. their RDase sequences did not comprise a single unique clade per OG), including OG91/128 (>91% identity), OG121/141 (>90%), OG102/152 (>91%), OG134/138 (>94%), OG125/163 (>93%), OG159/137 (>91%), OG108/160 (>99%), OG100/7 (>97%) and OG99/45 (>93%). These OGs were merged for visualization moving forward, maintaining their former designation (i.e. OG102, in OG102/152). Singletons (sequences sharing <90% identity with any other RdhA) were assigned a letter designation (A–Q). These sequences belong predominantly to strains HCH1 and 4CP – the two strains most distantly related to the other species of *Dehalobacter*. These singletons branch deeply in the phylogenetic tree next to common *Dehalobacter* OGs, such as singleton H next to OG87 (85% identity), A and OG142 (86% identity), G and OG343 (81% identity) or E and OG116 (83% identity), which is consistent with the distant relation of strains HCH1 and 4CP to the other *Dehalobacter* strains (Fig. 4). These ‘singleton umbrellas’ were considered when analysing interspecies synteny.

**Fig. 5.**
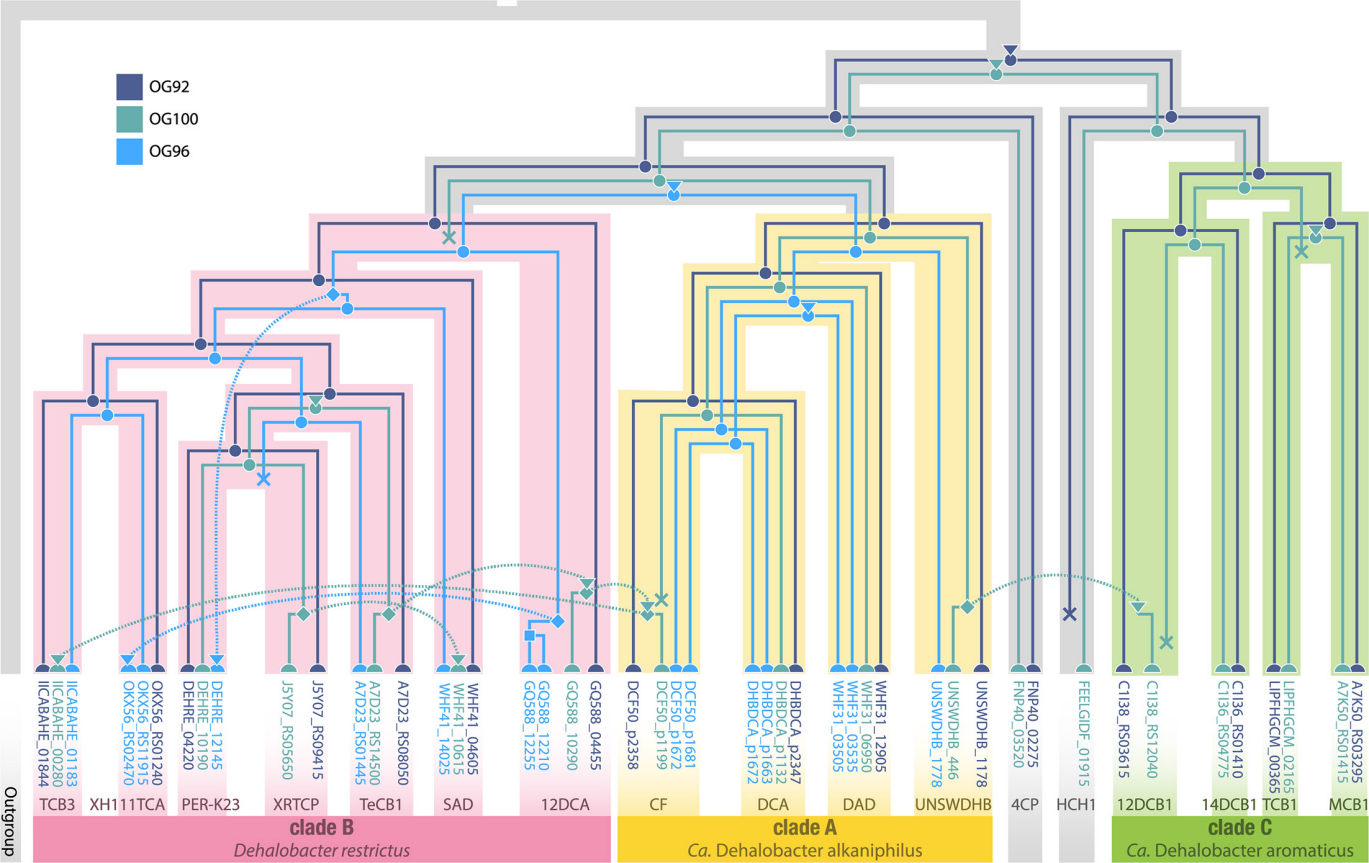
Reconciled species-aware phylogenetic gene tree of selected OGs. Each reconciled RDase gene tree is mapped inside the *Dehalobacter* species tree, which is coloured by clade. Each gene tree begins with an acquisition event represented by a downward triangle and has a symbol at each node representing an evolutionary event; speciation nodes are represented as circles, duplication nodes are squares and gene losses are crosses. Horizontal transfers are dotted lines starting from a diamond and ending with a downward triangular arrow.

No OG was present in all *Dehalobacter* genomes, but widespread OGs include OG92, OG101 and OG131. In the phylogenies of each of these common OGs, the RdhA sequences cluster according to *Dehalobacter* clade (i.e. closely related genomes have more similar RdhA sequences; Figs 4 and S9). This suggests that these OGs are likely old to the *Dehalobacter* genus – diverging concurrently with the individual *Dehalobacter* lineages. An example of this simultaneous divergence is shown in a species-tree aware gene phylogeny (Fig. 5) predicted by GeneRax [51], wherein a phylogenetic gene tree of OG92 (dark violet) is inset within a *Dehalobacter* species tree (shaded by clade/species). Initial acquisition of OG92 is predicted only once in a pre-speciation *Dehalobacter* ancestor (marked with a downward triangle), after which it has evolved with each lineage (each speciation node is marked with a circle). The only strain that no longer harbours an OG92 RDase is strain HCH1 (gene loss is marked with an X), whose genome is quite divergent from that of other strains. This lack may be due to gene loss over time, or an incomplete genome, as none of the genomes from which OG92, OG101 and OG131 are missing are closed.

Conversely, other common OGs, like OG100, comprise very similar sequences that cluster independently of the *Dehalobacter* clade from which they were extracted, which may indicate HGT rather than vertical inheritance (Fig. S9). Accordingly, GeneRax predicts that OG100 has independently arisen in eight lineages of *Dehalobacter* (Fig. 5, teal triangles), with at least five predictions of HGT within these select genomes. The complete phylogenetic history of highly transferred OGs is challenging to resolve, as acquisition has certainly occurred from organisms external to this pangenome.

Horizontal transfer of an OG96 RDase operon (*pceABCD*) was previously proposed in *Dehalobacter* strains PER-K23 and CF/DCA due to the presence of cryptic transposase genes or inverted repeats on either side of the gene cassette [25,26]. Unexpectedly, the OG96 sequences do cluster by *Dehalobacter* clade, which typically suggests sequence divergence during species evolution rather than HGT (Fig. S9). The reconciled species-gene tree for OG96 (Fig. 5, true blue) predicts that OG96 initially arose in *Dehalobacter* prior to the split in clades A and B, with several instances of HGT (dashed lines; ex: strain PER-K23 to XH111TCA) and duplication (squares; ex: strain 12DCA). Interestingly, the strains in which HGT of OG96 was previously proposed each show sequence diversity compared to others in their clade; our work predicts HGT of OG96 in strain PER-K23 (Fig. 5) and the second copy of OG96 in strains CF and DCA (in Fig. 5, the acquisition event immediately prior to branching of the DAD lineage). This species-aware sequence-based HGT prediction therefore aligns with the genomic architecture-based approaches of prediction in prior work.

### RDase cluster synteny in *Dehalobacter* genomes

**Fig. 6.**
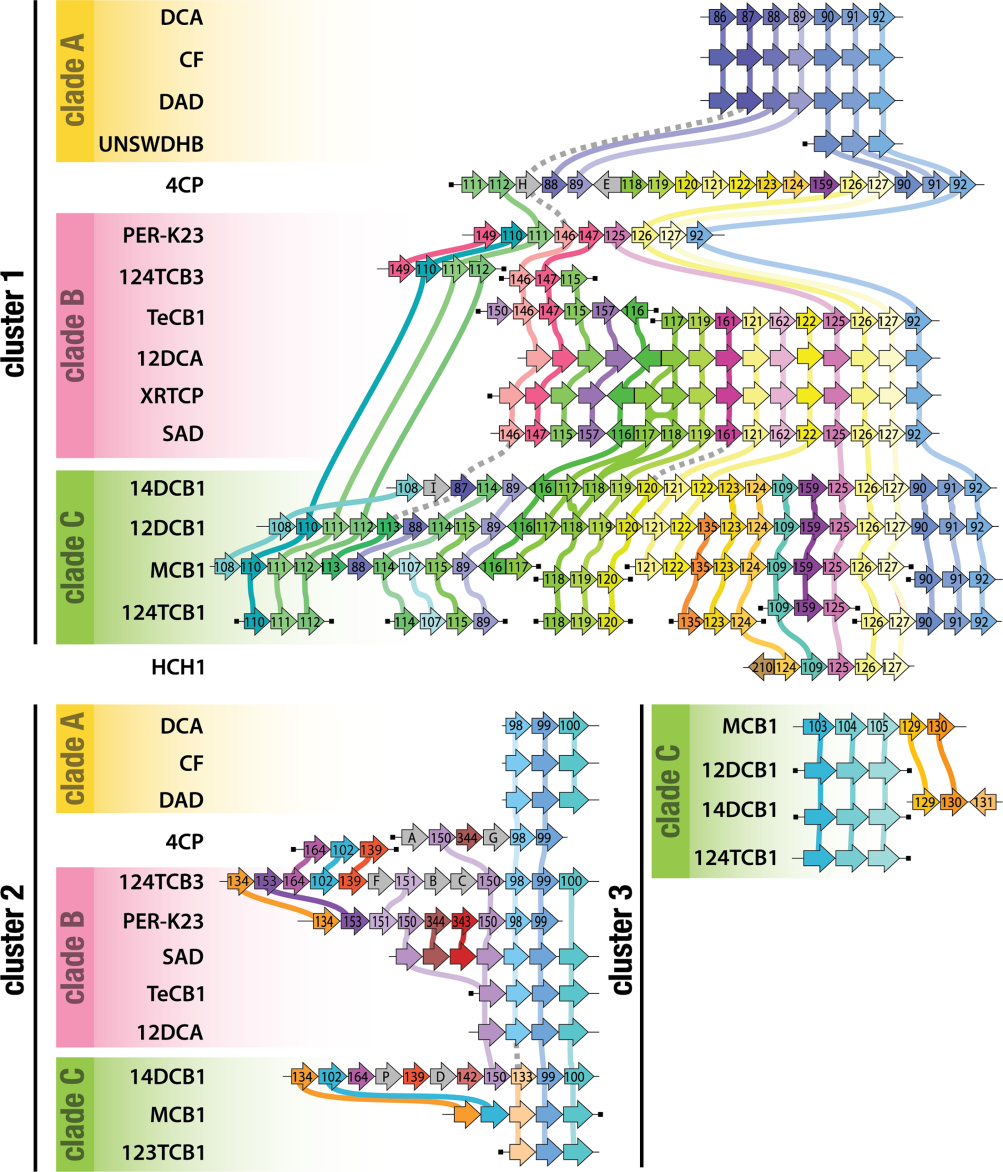
Alignment of concatenated adjacent RdhA sequences in RDase clusters. Each RDase is coloured by OG as in Fig. 4. Grey RDases represent singleton sequences (i.e. not a part of any OG). Solid lines represent >90% aa identity (RDases within the same OG); grey dashed lines represent connections between OG/singleton umbrellas (75–90% similarity). Each clade of *Dehalobacter* is demarcated by colour.

In each complete *Dehalobacter* genome, there are two distinct clusters of many closely localized RDases, as well as several genomically isolated RDases (seven to eight in clade A and three to four RDases in clade B) that may be a vestige of genome rearrangements or HGT. The *rdhA* sequences from each RDase cluster were concatenated, and the strings of RDase sequences were aligned to investigate the degree of RDase synteny across *Dehalobacter* genomes (Fig. 6). The intervening sequences removed for visualization included mainly *rdhBC* genes, as well as transposases and transcriptional regulators (Fig. S7). The alignments between RDase clusters from closed genomes were bolstered with concatenated *rdhA* sequences from the contigs in multi-contig MAGs. No adjacent RDase sequences could be found within the genomes of strains 124TCB3 or XH111TCA due to their fragmentation, so they are not visualized. High-density RDase regions can be difficult to assemble due to many repeated sequences that can lead to assembly breaks, and many of the RDase clusters extracted from multi-contig MAGs were found at the ends of contigs (denoted with a black square on the end of a cluster in Fig. 6). These likely only represent a slice of the full RDase cluster, but even cluster subsections are useful for supporting the synteny we see between the complete clusters extracted from circular genomes.

Cluster 1 is much larger than cluster 2, especially in clades B and C (*D. restrictus* and *Ca*. D. aromaticus), in which cluster 1 contains ~15 RDases and ~25 RDases, respectively. Clade A (*Ca*. D. alkaniphilus) has fewer RDases overall, and cluster 1 is limited to three to seven RDases. The synteny within RDase clusters is clear across genomes from each *Dehalobacter* clade (Fig. 6). Though cluster 2 is less conserved across clades, both clusters are most diverse towards their 5′ end. OG92 is common to the 3′ end of cluster 1 in all strains except HCH1, but the OG order upstream of OG92 is clade dependent. Working upstream, clades A and C share OG92-OG91-OG90 in all strains, after which the pattern diverges between clades. Clade B lacks OG91-90; these genomes instead have a pattern of OG92-127-126-125. Interestingly, this same series of OGs follows OG90 in clade C, suggesting clade B once mirrored clade C prior to the loss of OG91-90 in the evolutionary past. Notably, the RDases in each of these OGs cluster by clade in the RDase phylogeny (Fig. 4), as represented by OG92 in the species-aware gene tree of Fig. 5, which supports their ancient acquisition and sequence divergence concurrent to speciation.

Moving towards the 5′ end of the clusters, there continue to be many similarities between clades B and C, with extra OGs interspersed in clade C (ex: OG123-124-109-159 between OG125 and OG122). This could be due to either (1) gene duplication and divergence in clade C or (2) genome streamlining and subsequent loss of RDases in clade B. If this was truly due to clade-specific gene duplication, we would not expect strain 4CP to carry any of these OGs, since it is more phylogenetically distinct from clade C than clade B; however, we do in fact see OG123-124-159 in strain 4CP. Similarly, OG111-112 is common to clade C and strain 4CP, but missing in clades A and B. Within clade B, strain PER-K23 has also lost a nine-RDase region between OG147 and OG125 (OG115-157-116-117-119-161-121-162-122) that is common to the other closed genomes in its clade. Altogether, these observations suggest that gene loss may be a more major differentiator between RDase OG discrepancies than gene duplication and divergence in cluster 1, which may have been obtained simultaneously through an ancient acquisition event prior to speciation within the *Dehalobacter* genus.

RDase synteny across *Dehalobacter* clades can be seen even more clearly when considering similarity beyond the confines of an OG. OGs are defined, somewhat arbitrarily, as sharing more than 90% identity, and are further curated based on phylogenomic tree clades. If we expand past a single clade, looking one node deeper towards the centre of the tree (where all leaves are still >75% similar), more connections can be seen across clades. For example, OG161 and OG120 are closely related on the phylogenetic tree (>85% identity), but OG161 only contains sequences from strains in clade B (Fig. 4). Conversely, OG120 contains sequences from strains in clade C and strain 4 CP (Figs 6 and S9). If we consider these OGs under one OG umbrella, the degree of inter-clade synteny increases, as OG161/120 is always located between OG119 and OG121 (Fig. 6, connected with dashed grey lines). The same effect can be seen in umbrellas for OG98/133 (>77% identity), OG108/149 (>87%) and OG87/146/113 /H (>76%) (Fig. 6, connected with dashed grey lines). In these cases, clade-specific sequence divergence is responsible for OG differentiation, but the RDases within are tied to a shared evolutionary history.

Several gene duplications can also be seen, such as in the case of OG118/117 in clades B and C – some sequences between which still share >90% identity (Fig. 6, cluster 1; Fig. S9) – as well as OG150 in clade B (Fig. 6, cluster 2). Interestingly, two members of clade B (strains 12DCA and TeCB1) avoided both duplication events, unlike the rest of the clade. It is clear that RDase clusters are influenced by a number of factors and are indicative of a genus adapting to a changing environment, especially as anthropogenic chemicals expand the breadth of available electron acceptors usable for respiration.

### Each *Dehalobacter* species favours one RDase OG

Though we lack RDase characterization data from all selected *Dehalobacter* strains, the published data indicate an interesting pattern of RDase usage. All strains in clade A (*Ca*. D. alkaniphilus) express an OG97 RDase, each of which dechlorinates structurally related chlorinated alkanes as preferential substrates (Table 1). The active RDases of four strains in clade B (*D. restrictus*) have been characterized, three of which belong to OG96 despite dechlorinating various substrates (chlorinated alkanes, alkenes and aromatics; Table 1). Strain PER-K23 uses PceA to dechlorinate PCE to *cis*-DCE [5]. Strains TeCB1 and 124TCB3 express TcbA and TcbA_KB1TCB3, respectively, which share >95% aa identity with PceA but dechlorinate tetra- and tri-chlorobenzenes [8,60] (Table 1). Of the uncharacterized strains, all clade B genomes encode an OG96 RDase except the fragmented genome of strain XRTCP. Clade C (*Ca*. D. aromaticus) currently only encompasses strains that respire chlorinated aromatics. Strain MCB1 is known to express McbA (OG102), and strains 12DCB1 and 14DCB1 also express OG102 RDases (Steve Zinder, personal communication). Strain HCH1 uses HchA (OG145) to dechlorinate (β-HCH) to monochlorobenzene (MCB) [60].

**Table 1.** Active RDases and their OG from each characterized strain in the *Dehalobacter* pangenome

Clade	Strain	Active RDase	OG	Primary activity (in culture)	Reference
A	CF	CfrA	97	CF to DCM; 1,1,1-TCA to 1,1-DCA	[65]
	DCA	DcrA	97	1,1-DCA to CA	[65]
	UNSWDHB	TmrA	97	CF to DCM; 1,1,2-TCA to 1,1-DCA and VC	[66]
	DAD	BcdA	97	CF to DCM	[28]
B	PER-K23	PceA	96	PCE to *cis*-DCE	[5]
	TeCB1	TcbA	96	1,2,4,5-TeCB to 1,2,4-TCB;1,2,4-TCB to 1,3-DCB and 1,2-DCB	[8]
	124TCB3	TcbA_KB1TCB3	96	1,2,4-TCB to 1,3-DCB and 1,4-DCB	[60]
	SAD	AcdA	97	CF to DCM	[28]
C	MCB1	McbA	102	MCB to benzene	Stephen Zinder,unpublished data
	12DCB1	Unnamed	102	1,2-DCB to MCB	
	14DCB1	Unnamed	102	1,4-DCB to MCB	
na	HCH1	HchA	145	β-HCH to MCB	[60]

CA, chloroethane; DCB, dichlorobenzene; DCE, dichloroethene; MCB, monochlorobenzene; TCA, trichloroethane; TCB, trichlorobenzene; TeCB, tetrachlorobenzene; VC, vinyl chlorideβ-HCHβ-hexachlorocyclohexane

Of all the active RDases, only OG102 is part of either RDase cluster, while others are encoded extraneously in the genome – perhaps indicative of HGT in a selective environment. The RDase expression patterns and electron acceptor specificity within each clade suggest an evolutionary relationship between RDase use and speciation in the genus, but RDase characterization in additional strains is critical to fully understand this phenomenon. Moreover, the collection of available *Dehalobacter* genomes is biased in their association with contaminated sites; natural ‘non-anthropogenic’ substrates are sorely missing from our understanding of the genus’s history.

*D. restrictus* SAD is seemingly exempt from this OG–clade relationship; it expresses an OG97 RDase like clade A, despite also encoding an OG96 RDase in its genome like other clade B strains [28]. This RDase – named AcdA – is not found in either RDase cluster but rather sits upstream of the *mec* cassette, which encodes ten genes involved in DCM degradation [28]. Strain SAD was the first *D. restrictus* (clade B) genome found to harbour the *mec* cassette, though all known *Ca*. D. alkaniphilus (clade A) genomes encode at least a partial *mec* cassette. Interestingly, strain XH111TCA is another haloalkane dechlorinating strain in clade B whose genome encodes an OG97 RDase (description found in deposited BioSample: SAMN31436522). We identified a *mec* cassette downstream of this RDase, which is syntenic with strain SAD and the clade A (*Ca*. D. alkaniphilus) strains (Fig. S8). Moreover, the MAG of an additional 1,1,1-TCA and CF-dechlorinating strain – *D. restrictus* 8M (clade B) – was deposited on the MicroScope platform (ID: 14849), and in it, we identified a homologous OG97-*mec* cassette genomic neighbourhood (Fig. S8). In clade B, this region is specific to the chloroalkane-degrading minority but is syntenic to the OG97*-mec* neighbourhoods in strain DAD and the other clade A strains, which prompted a deeper investigation of the origin of this uncharacteristic clade B phenotype.

### An RDase-encoding genomic island is common to SC05 *Dehalobacter* genomes

Though their overall median ANI is only 95.3%, the genomes of the two *Dehalobacter* strains in the SC05 subcultures – strains SAD and DAD – share one common ~143 kb region with 98.7% sequence identity, suggesting transmission via HGT. This shared region includes the OG97 RDase and *mec* cassette, as part of a larger 370 kb region (marked in blue in Figs1  3). PacBio HiFi long-read coverage over each end of this region supports its integration into each *Dehalobacter* genome, as well as its circularization when excised. This circular element lacks an origin of replication, which is indicative of a large MGE rather than a plasmid. The integrated and circularized forms of the MGE were both detected via site-specific PCR and Sanger sequencing in SC05-UT and DCME (expanded upon in Text S4).

To delimit this putative MGE in each *Dehalobacter* genome, the nearest core genes to each end of the region were selected from the pangenome. The delimited genomic island begins at the 5′ end of the last *rdhB* gene in cluster 2 (DAD: OG98 and SAD: OG150; Fig. S7) and ends at the 3′ end of an RNA methyltransferase gene (*rumA*). No gene in the 100–400 kb between these two bookends is shared among the six closed *Dehalobacter* genomes. Multi-contig genomes were excluded from this analysis, as their fragmented nature precludes alignment and delimitation. The 3′ end of *rumA* is an established insertion site for several types of MGEs, including transposons and larger (60–80 kb) integrative chromosomal elements (ICEs) encoding serine recombinases, marking this location of the *Dehalobacter* genome as an integration hotspot [61,63].

The possible implication of the *rdhB* gene in MGE integration is unknown but could indicate a route of integration for RDase HGT in the past, especially considering that the 5′ end of RDase cluster 2 (i.e. the end exposed to the MGE) lacks the synteny of the 3′ end (Fig. 6). The integration and excision of mobile elements may facilitate addition and removal of various RDases to this cluster in the genome, including the active RDase in clade C (OG102, Fig. 6). This method of acquisition would not, however, affect those RDases in cluster 1, which exists between many contiguous core genes on either side.

### An integration hotspot in the *Dehalobacter* genus

**Fig. 7.**
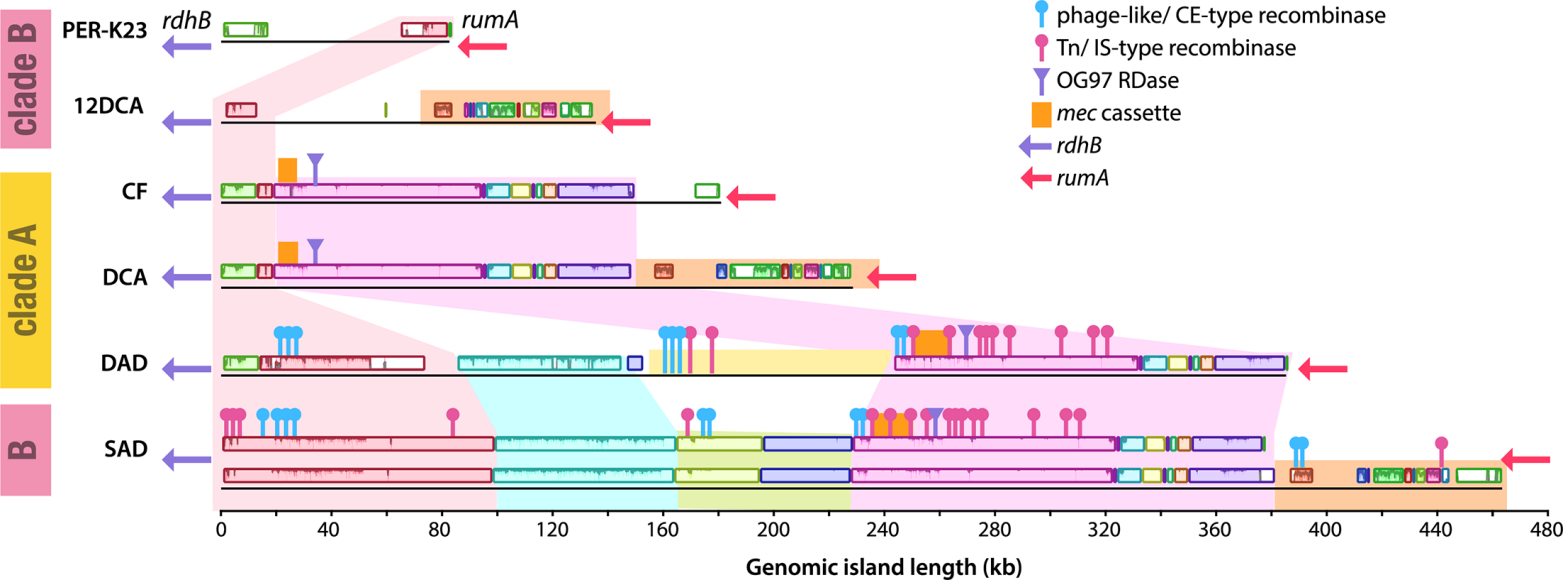
Alignment of nested recombinase island extracted from all closed *Dehalobacter* genomes from *rdhB* to *rumA*. Each coloured rectangle represents an LCB, and highlighted regions represent consistently consecutive blocks. CE, conjugative element; Tn, transposon; IS, insertion sequence.

To further evaluate this genomic island, regions between *rumA* and the *rdhB* from each closed *Dehalobacter* genome were extracted and aligned. Unlike the full genomes and RDase clusters, these aligned regions are not easily identifiable by clade – indicative of a region influenced by HGT. The alignment reveals several distinct colinear blocks common to multiple *Dehalobacter* species (Fig. 7). Each of these motifs is explored in Text S6.

Multiple recombinases existing between two core genes – nested recombinase islands – are difficult to disentangle due to the diversity of MGEs; most genes in these regions are annotated as hypothetical proteins [58]. Nonetheless, the number and classification of MGEs in this nested island in each closed genome were determined using the ProMGE database [58] (available in Text S5). This genomic island hosts 10–14 recombinases in strains CF, DCA and PER-K23 (Table S4). The number and type of nested recombinase islands were also determined in the SAD and DAD *Dehalobacter* genomic islands (Table S5). In the strain SAD genomic island, 28 recombinases were detected; 18 were classified as transposon/insertion sequences (Fig. 7, pink), and 10 were classified as conjugative elements/mobility islands/phage-like recombinases (Fig. 7, blue). In the strain DAD genomic island, 19 recombinases were detected (11 transposons/insertion sequences and 8 conjugative elements/phage-like recombinases). The differences in modules between the two nested recombinase islands, despite the high per cent identity, indicate that HGT of this region likely occurred longer ago than during lab enrichment.

Despite the inability to delimit and classify each internal element within this hotspot, this region of the *Dehalobacter* pangenome undoubtedly consists of a series of integrated mobile elements, including the remnants of many insertion sequences, transposons, ICEs and phages. This phenomenon is consistent across all closed *Dehalobacter* genomes and provides a putative mechanism of acquisition for RDases and other functional proteins, such as the *mec* cassette.

### Distinct *Dehalobacter* populations in the SC05 subcultures

One *Dehalobacter* contig was previously found to encode the OG97 RDase and *mec* cassette in both SC05 subcultures, determining one *Dehalobacter* strain was capable of CF dechlorination and DCM mineralization in SC05 [28]. Here, we determined that this contig is a large MGE integrated into the genomes of two distinct *Dehalobacter* strains – SAD and DAD. Though each genome was extracted from a different subculture of SC05 (SC05-UT vs. DCME), these subcultures derive from the same parent culture, enabling the presence of both strains in each culture. Therefore, it is possible that either or both *Dehalobacter* strains perform CF dechlorination and DCM mineralization in each subculture. In previous studies of SC05, only one *Dehalobacter* was detected in any subculture by 16S rRNA-specific qPCR [29], but the rRNA sequences of each strain (SAD and DAD) are identical in the V6–V8 region, which was previously used for this detection (Fig. S4). All four of strain SAD’s rRNA sequences share 99.8–100% identity, while in strain DAD, only two of three rRNA genes share 99.9% identity. The third strain DAD rRNA gene more closely resembles strain SAD’s rRNA sequence (94.1% identity, compared to 99.1% identity), which makes strain-specific detection and quantification challenging using the 16S sequence (Fig. S4).

**Fig. 8.**
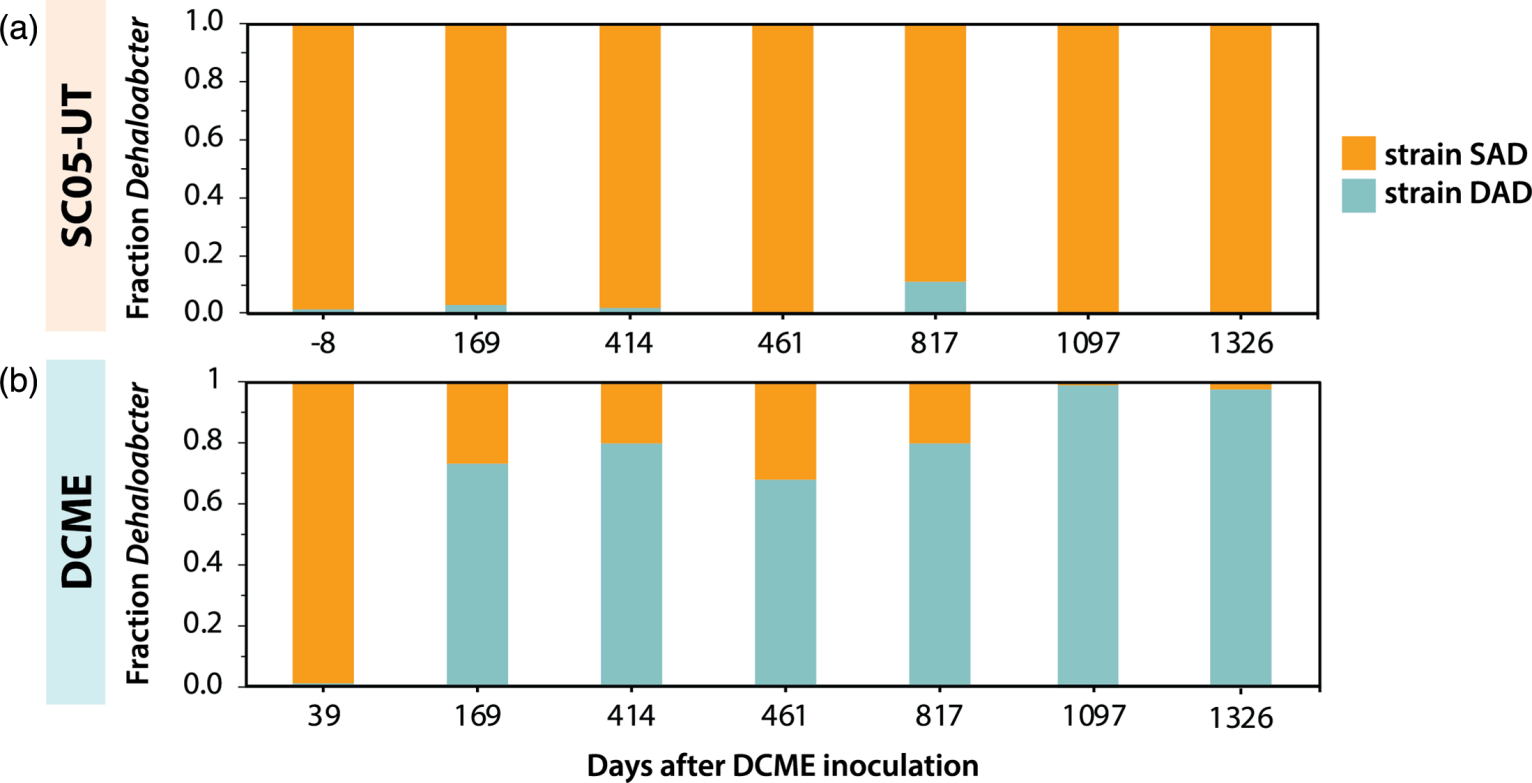
Relative strain composition of *Dehalobacter* population in (a) SC05-UT and (b) DCME during enrichment (T_0_=DCME inoculation, Fig. S1). Strain SAD is in orange, and strain DAD is in blue.

To determine the presence of each *Dehalobacter* strain in each SC05 subculture (SC05-UT vs. DCME), a single-copy core gene with high variation between *Dehalobacter* genomes was selected for qPCR: a flagellar basal body protein (*flgC*). The abundance of each strain was measured in DNA samples taken throughout the enrichment of each culture (Fig. 8). The *Dehalobacter* population of SC05-UT was composed of >97% strain SAD, except day 817, when strain DAD rose to 11% abundance. DCME initially reflected the population of its parent culture (SC05-UT), but during sequential feeding of DCM alone, strain DAD increased to 75% by day 169 and eventually comprised >97% of the DCME *Dehalobacter* population. The metagenomes of each culture were first assembled from samples extracted on days 414 and 461, from which only a poor-quality *Dehalobacter* MAG was assembled from DCME [35]. This may have been due to assembly errors caused by the occurrence of both *Dehalobacter* strains at >15%. When additional samples were sequenced on day 1097, strain DAD made up 98.9% of the *Dehalobacter* population, allowing a more complete assembly. Whether the DCME conditions were especially favourable for strain DAD, or especially hostile to strain SAD, or whether stochastic events caused this change in population dynamics are yet to be known.

### Implications for *Dehalobacter* evolution

With the addition of two new closed *Dehalobacter* genomes – *D. restrictus* SAD and *Ca*. Dehalobacter alkaniphilus DAD – to the growing database, pangenomics allows definition of three clear multi-genome clades within the *Dehalobacter* genus. The results of this genome analysis strongly suggest that strains currently classified as *D. restrictus* belong to one of the five distinct lineages that could be classified as species – one for each of clade A (*Ca*. Dehalobacter alkaniphilus), clade B (*D. restrictus*) and clade C (*Ca*. Dehalobacter aromaticus), along with recognition of strains 4CP and HCH1 as individual species themselves. Additional species are likely to resolve as the collection genomic data within this genus grow. Though phenotype (the active RDase’s sequence classification into OG and its substrate preference) may also be indicative of *Dehalobacter* species, some strains have acquired an alien RDase by interspecies HGT, influencing their phenotype. The discovery of this MGE encoding an RDase and the *mec* cassette indicates the transfer of function – namely, CF dechlorination and DCM mineralization – between *Dehalobacter* species.

This analysis contributes to a growing understanding of the evolutionary history of the *Dehalobacter* genus, particularly regarding adaptation to anthropogenic chemicals. Conserved RDase patterns suggest that loss of RDases over time is a major determinant of function, but this may be reflective of the nature of *Dehalobacter* research. There is undoubtably a selection bias pertaining to the studied strains of *Dehalobacter*; these organisms are studied mainly in the context of bioremediation and are often enriched on an anthropogenic chemical of interest to which their ancestors would not have had ample access. The multitude of RDases in each *Dehalobacter* genome may have once been necessary to dechlorinate an assortment of naturally occurring organohalides, like those produced from volcanoes, fires, geothermal processes or biologically by algae, sponges and other marine and terrestrial life [64]. Loss of RDases over time may be directly resultant of increased access to large sources of man-made organohalides and our methods of cultivation, which may be elucidated with increased screening for *Dehalobacter* and related lineages in pristine environments.

#### Description of *Candidatus Dehalobacter alkaniphilus* sp. nov.

*Candidatus* Dehalobacter alkaniphilus (al.ka’ni.phil.lus. L. masc. n. *alkanum* alkane; Gr. adj. *philus* loving; N.L. masc. adj. *alkaniphilus* alkane-loving)

The member of the *Dehalobacter* genus, *Candidatus* Dehalobacter alkaniphilus, is a bacterial species identified by metagenomic analyses. The genome length of the type genome is 3 069 953 bp and the G+C content is 44.61%.

The type material is GCF_000305775.1, a MAG describing *Dehalobacter* strain DCA from a groundwater-derived enrichment culture.

#### Description of *Candidatus Dehalobacter aromaticus* sp. nov.

*Candidatus* Dehalobacter aromaticus (ar.o.ma’ti.cus. L. masc. adj. *aromaticus* aromatic fragrant; referring to its ability to dechlorinate aromatic compounds)

The member of the *Dehalobacter* genus, *Candidatus* Dehalobacter aromaticus, is a bacterial species identified by metagenomic analyses. The genome length of the type genome is 2 850 914 bp and the G+C content is 44.38%.

The type material is GCF_004343605.1, a MAG describing *Dehalobacter* strain 12DCB from a pure culture derived from a drainage ditch in NJ, USA.

## supplementary material

10.1099/mgen.0.001324 Uncited Supplementary Material 1.

10.1099/mgen.0.001324 Uncited Supplementary Material 2.

## Abbreviations

ANI: average nucleotide identity
CF: chloroform
DCA: dichloroethane
DCM: dichloromethane
GTDB: Genome Taxonomy Database
HGT: horizontal gene transfer
ICEs: integrative chromosomal elements
IMG/M: Integrated Microbial Genomes and Metagenomes
LCB: locally colinear block
MAG: metagenome-assembled genome
MCB: monochlorobenzene
MGE: mobile genetic element
ML: maximum-likelihood
NCBI: The National Center for Biotechnology Information
OG: orthologue group
OHRB: organohalide-respiring bacteria
PCE: perchloroethene
qPCR: quantitative PCR
RDases: reductive dehalogenases
TCA: trichloroethane
β-HCH: β-hexachlorocyclohexane
