# Supplementary material for: Pangenomic insights into Dehalobacter evolution and acquisition of functional genes for bioremediation
Source: Microb Genom. 2024 Nov 20;10(11):001324. doi: 10.1099/mgen.0.001324 (PMC11578063; doi:10.1099/mgen.0.001324)
Supplement: Uncited Supplementary Material 1. [file mgen-10-01324-s001.pdf]

Supplemental Information

Pangenomic insights into *Dehalobacter* evolution and acquisition of functional genes for bioremediation

Olivia Bulka[a], Radhakrishnan Mahadevan[a], Elizabeth A. Edwards[a#]

[a]Department of Chemical Engineering and Applied Chemistry, University of Toronto, Toronto, Ontario, Canada

#Address correspondence to Elizabeth A. Edwards, elizabeth.edwards@utoronto.ca.

Table of Contents

Table of Contents ... 2
Supplementary Figures ... 2
Supplementary Tables ... 3
TEXT S1. Metagenome sequencing and genome curation ... 4
TEXT S2. Comparison of publicly available *Dehalobacter* strains ... 6
TEXT S3. *Dehalobacter* clade and RDase analysis ... 7
TEXT S4. Experimental detection of MGE and strain-specific PCR ... 13
TEXT S5. *Dehalobacter* mobilome ... 15
TEXT S6. A survey of the genomic island modules ... 20
SI References ... 22

Supplementary Figures

Figure S1. Schematic of metagenomic sequencing and assembly according to enrichment timeline ... 5

Figure S2. Example of read coverage after sequencing "Round 1" with PCR amplification ... 5

Figure S3. Phylogenetic tree of 16S rRNA genes from the selected *Dehalobacter* strains. ... 7

Figure S4. Alignment of 16S rRNA genes from *Dehalobacter* strains SAD and DAD. ... 8

Figure S5. Clade-specific gene clusters and their assigned COG20 category. ... 8

Figure S6. A closer inspection of three locally colinear blocks (LCBs) in the genome-scale alignment of six circularized *Dehalobacter* genomes. ... 9

Figure S7. Alignment of genes in RDase clusters from closed *Dehalobacter* genomes. ... 10

Figure S8. Alignment of the gene neighborhoods containing the OG97 RDase and *mec* cassette from *Dehalobacter* genomes. ... 11

Figure S9. Phylogeny of reductive dehalogenases in select Ortholog Groups ... 12

Figure S10. Targeted amplification of circularized and integrated MGEs in SAD and DAD genomes and primer design schematic ... 13

Figure S11. Gene structure of LCB motifs encoding putative DNA transfer systems in HGT region extracted from all closed *Dehalobacter* genomes. ... 20

Supplementary Tables

Table S1. Summary of genomically or experimentally characterized *Dehalobacter* strains prior to December 2023. .... *supplemental excel*

Table S2. Selected *Dehalobacter* genomes for pangenomic analysis 6

Table S3. Primer sequences for targeted amplification of circularized and integrated IMEs and genomes, as well as *flgC* strain-specific qPCR 14

Table S4. Detected recombinases in the nested genomic island of the *Dehalobacter* strains determined by proMGE 15

Table S5. Detected recombinases in the *Dehalobacter* strain DAD MGE and their classifications 15

Table S6. Conjugative and secretion systems detected in the genomes of *Dehalobacter* strains A) DAD and B) SAD and their classifications 17

Table S7. Putative prophage-derived regions in the *Dehalobacter* strain SAD and DAD genomes 19

TEXT S1. Metagenome sequencing and genome curation

Metagenome sequencing and assembly as well as curation of both *Dehalobacter* genomes are expanded upon in our related publication in Microbiology Resource Announcements (1), using PacBio reads from prior sequencing described in another related publication (2) (Figure S1). Sequencing Round 1 produced one 3.46 Mb *Dehalobacter* bin in 7 contigs from the SC05-UT metagenome, and one 1.97 Mb *Dehalobacter* bin in 28 contigs from the DCME metagenome—missing a significant amount of a typical *Dehalobacter* genome (~3–3.5 Mb) (3). Neither MAG could be closed, in part due to "wavy" read mapping resulting from PCR amplification (Figure S2). Round 2 resulted in a 1.31 Mb *Dehalobacter* bin in 805 contigs from the SC05-UT reads, and a 3.29 Mb *Dehalobacter* bin in 7 contigs from the DCME reads (1). The 805 contigs in the new SC05-UT bin were mapped to the Round 1 SC05-UT bin, and showed a 99.9% ANI, but were ultimately discarded due to their short length and redundant information. The longer DCME contigs, however, were further curated to represent the DCME *Dehalobacter* population.

The *Dehalobacter* strain SAD genome was closed with and without an additional ~100 kb segment at the 3' end of the integrated MGE. Both integration configurations could be detected via PCR, though the configuration with the extra segment saw much lower amplification (Figure S10). Additionally, the MGE-free genome could only be detected by PCR when the extra segment was included, suggesting the MGE could have replaced this segment, or vice versa, in some members of the population.

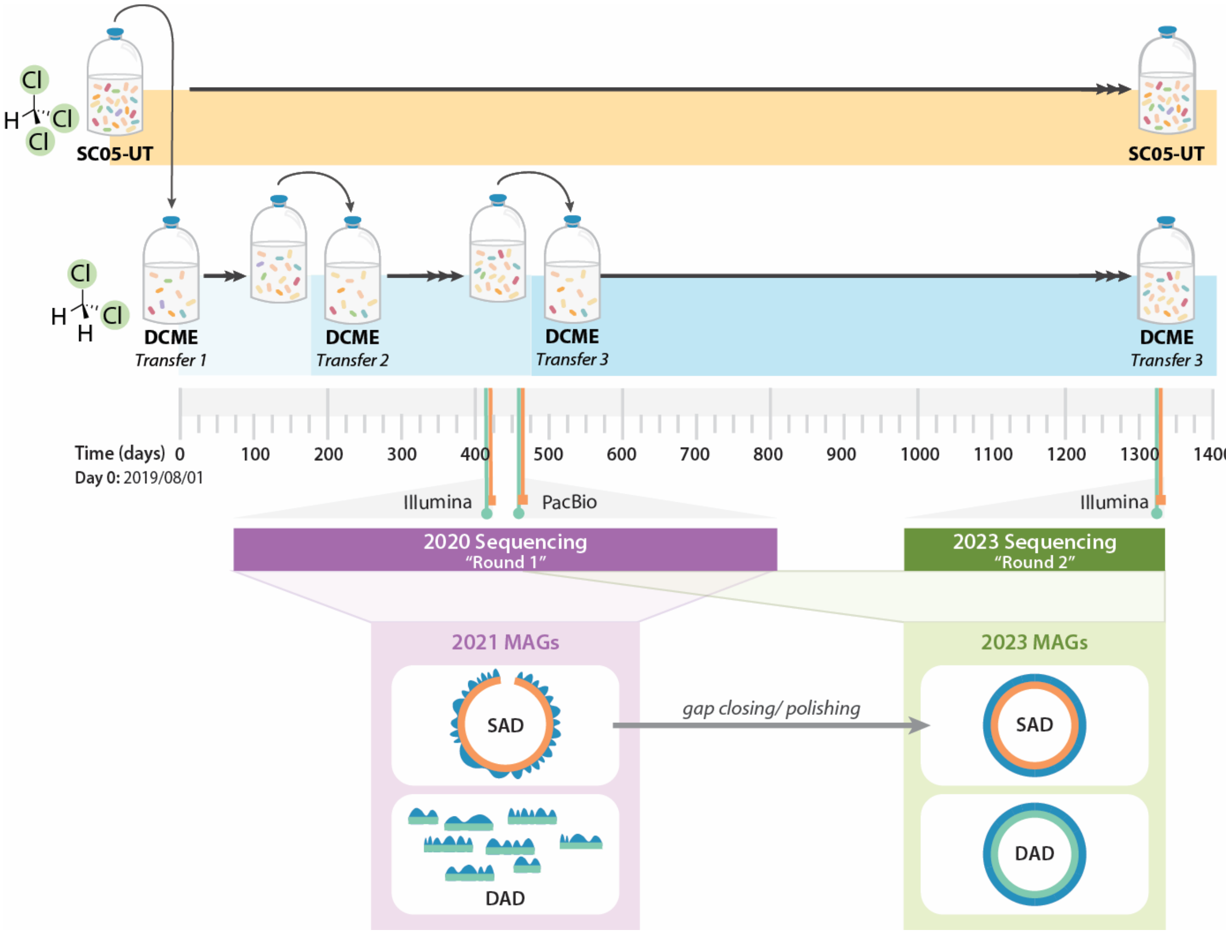

Figure S1. Schematic of metagenomic sequencing and assembly according to enrichment timeline. DNA samples are noted on the timeline (SC05-UT: orange squares, DCME: green circles), and labelled with type of sequencing and sequencing round. *Dehalobacter* MAG results from each round are depicted in the lower panels; *Dehalobacter restrictus* strain SAD (from SC05-UT): orange genome, *Dehalobacter* sp. strain DAD (from DCME): green genome; Example read mapping coverage shown in blue.

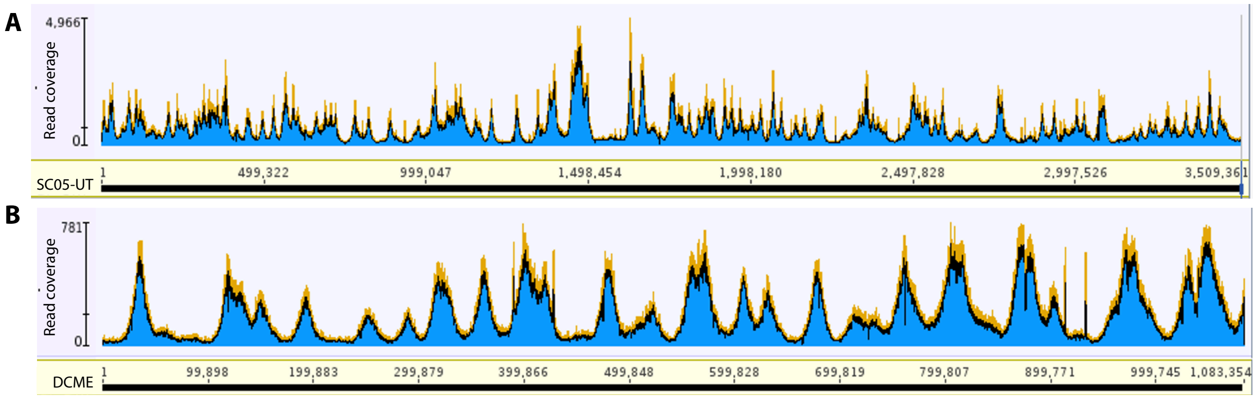

Figure S2. Example of read coverage after sequencing "Round 1" with PCR amplification on a contig from A) the SC05-UT culture, and B) the DCME cultures.

TEXT S2. Comparison of publicly available *Dehalobacter* strains

To create the *Dehalobacter* pangenome, the available literature and genomes were compiled and compared. A summary of characterized Dehalobacter strains can be found in the supplemental excel (Table S1), and the selected high-quality genomes are described in Table S2.

Table S1. Summary of genomically or experimentally characterized *Dehalobacter* strains prior to December 2023. *Described in supplemental excel.*

Table S2. Selected *Dehalobacter* genomes for pangenomic analysis, their CheckM completion and contamination (c_Clostridia marker lineage), and their assigned species according to the Genome Taxonomy Database R220. NA = not applicable.

**Strain**	**Mb**	**Completion (%)**	**Contamination (%)**	**Database**	**Assembly Accession**	**Genbank**	**Ref.**	**GTDB taxonomy (R220)**
DCA	3.1	99.94	0.00	NCBI	ASM30577v1	GCA_000305775.1	(4)	s__*Dehalobacter* sp000305775
CF	3.1	99.94	0.00	NCBI	ASM30581v1	GCA_000305815.1	(4)	s__*Dehalobacter* sp000305775
UNSWDHB	3.2	99.94	0.38	NCBI	UNSWDHB	GCA_000445165.1	(5)	s__*Dehalobacter* sp000305775
PER-K23	2.9	99.94	0.47	NCBI	ASM51289v1	GCA_000512895.1	(6)	s__*Dehalobacter restrictus*
12DCA	3	99.43	0.00	NCBI	ASM979630v1	GCA_009796305.1	(7)	s__*Dehalobacter restrictus*
TeCB1	3.1	99.94	0.17	NCBI	ASM170745v1	GCA_001707455.1	(8)	s__*Dehalobacter restrictus*
XRTCP	2.8	99.94	0.00	NCBI	ASM2199175v1	GCA_021991755.1	undescribed	s__*Dehalobacter restrictus*
XH111TCA	3	99.94	0.26	NCBI	ASM3006300v1	GCA_030063005.1	undescribed	s__*Dehalobacter restrictus*
MCB1	2.9	99.94	0.04	NCBI	ASM359027v1	GCA_003590275.1	undescribed	s__*Dehalobacter* sp004343605
12DCB1	2.9	99.94	0.04	NCBI	ASM434360v1	GCA_004343605.1	undescribed	s__*Dehalobacter* sp004343605
14DCB1	3	100.00	0.34	NCBI	ASM434363v1	GCA_004343635.1	undescribed	s__*Dehalobacter* sp004343605
4CP	3.4	99.94	7.57	NCBI	ASM991079v1	GCA_009910795.1	undescribed	s__*Dehalobacter* sp009910795
SAD	3.4	99.94	0.77	NCBI	UT-SAD-23	GCF_040500375.1	this study	NA
DAD	3.3	99.94	0.77	NCBI	UT-DAD-23	GCA_040500365.1	this study	NA
HCH1	2.6	99.94	0.17	IMG	2823892816		(9); *aka GT1; aka BinHCHmb12*	NA
124TCB1	2.7	99.94	0.04	IMG	2786546105		(9); *aka KB1_124TCB1*	NA
124TCB3	3.2	99.94	1.66	IMG	2821530269		(9); *aka BinKB1TCBm1*	NA

TEXT S3. *Dehalobacter* clade and RDase analysis

A phylogenetic tree was created to assess the relationship of *Dehalobacter* clades A and B in comparison to other strains. Complete clade B genomes each have 4 16S rRNA genes, all sharing >99.7% identity (Figure S3). Clade B genomes each have 3 16S rRNA genes, two of which are highly similar (>99.9% identity). The third rRNA gene is truncated at the 5' end, and is thus less similar to the other two sequences (~94% identity, visualized in Figure S4). The Clade B rRNA sequences only share 92-93% identity with the two non-truncated Clade A sequences, indicative of speciation. The sequences extracted from Clade C genomes are all only partial, rendering percent identity irrelevent. Nonetheless sequences >1 kb were included for visualization.

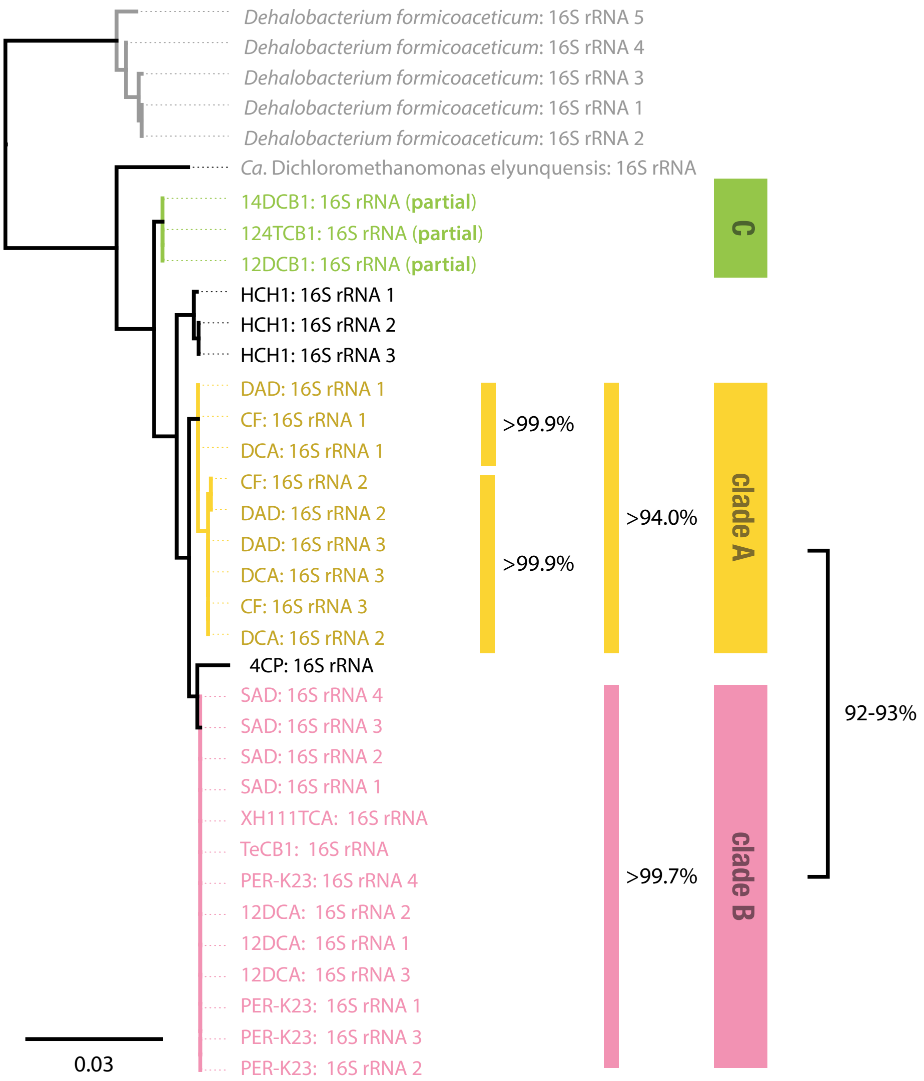

Figure S3. Phylogenetic tree of 16S rRNA genes from the selected *Dehalobacter* strains. Outgroups include *Ca.* Dichloromethanomonas elyunquensis and *Dehalobacterium formicoaceticum*. Percent identity is shown within and between Clades A and B. Scale bar in substitutions per site.

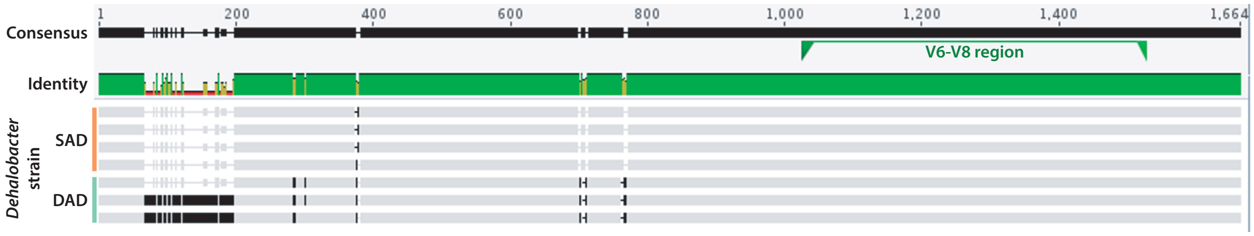

Figure S4. Alignment of 16S rRNA genes from *Dehalobacter* strains SAD and DAD. Primers used to amplify the V6-V8 region via amplicon are highlighted.

To further assess the speciation between Clades A and B, clade-specific gene clusters were extracted, assigned a COG category, and compared (Figure S5). Approximately half of the gene clusters unique to each clade did not have a corresponding COG category, but of those that did, Clade A is enriched for carbohydrate transport and metabolism genes, including fumarate reductase, methionine synthase, and cysteine lyase, which suggest a more complete TCA cycle and amino acid metabolism than other clades. Clade B is enriched in mobilome-specific—mainly transposases—and transcription-related gene clusters, as well as inorganic ion transport and metabolism compared to other clades. It also lacks the nitrogen fixation cassette present in Clades A and C. Clade C is enriched for cell wall and membrane biogenesis and signal transduction gene clusters. Functional enrichment cannot be quantified with the relatively low number of genomes currently available, but as additional *Dehalobacter* strains are added to the pangenome, more insights can be detected from this type of analysis.

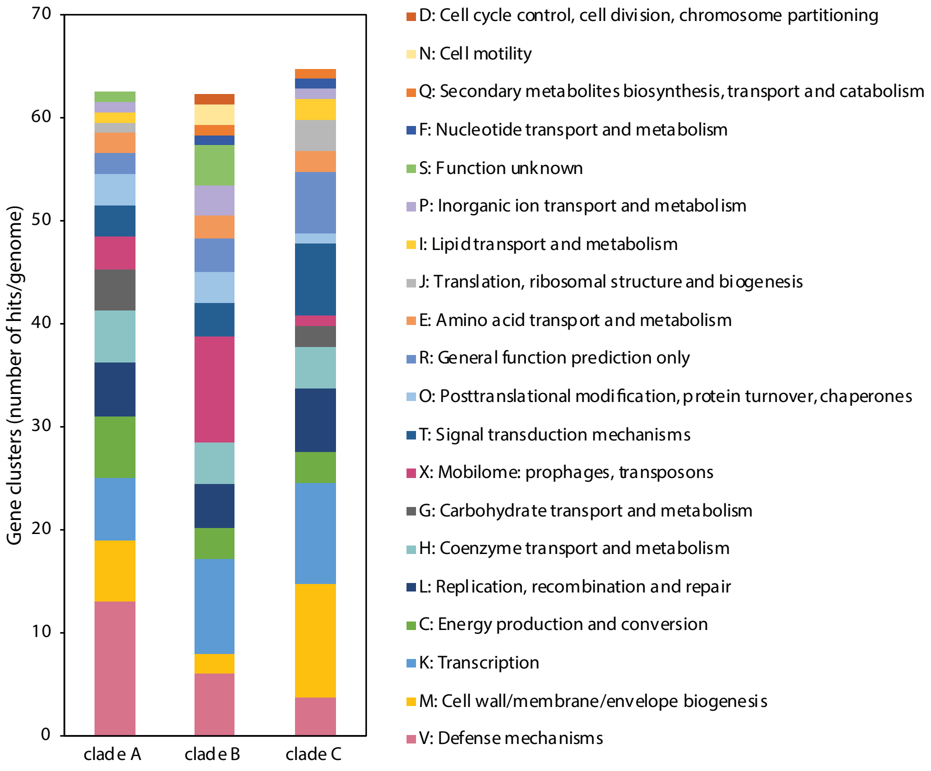

Figure S5. Clade-specific gene clusters and their assigned COG20 category.

Additionally, the locally colinear blocks (LCBs) pertaining to subsections of the aligned closed *Dehalobacter* genomes (Figure 3) were scrutinized. Many of these LCBs, though sufficiently syntenic to qualify as colinear, show visible variation across clades (Figure S6). Three examples were extracted for visualization, in which clear differences in terms of gene insertions and differential alignement can be seen when comparing Clade A to Clade B.

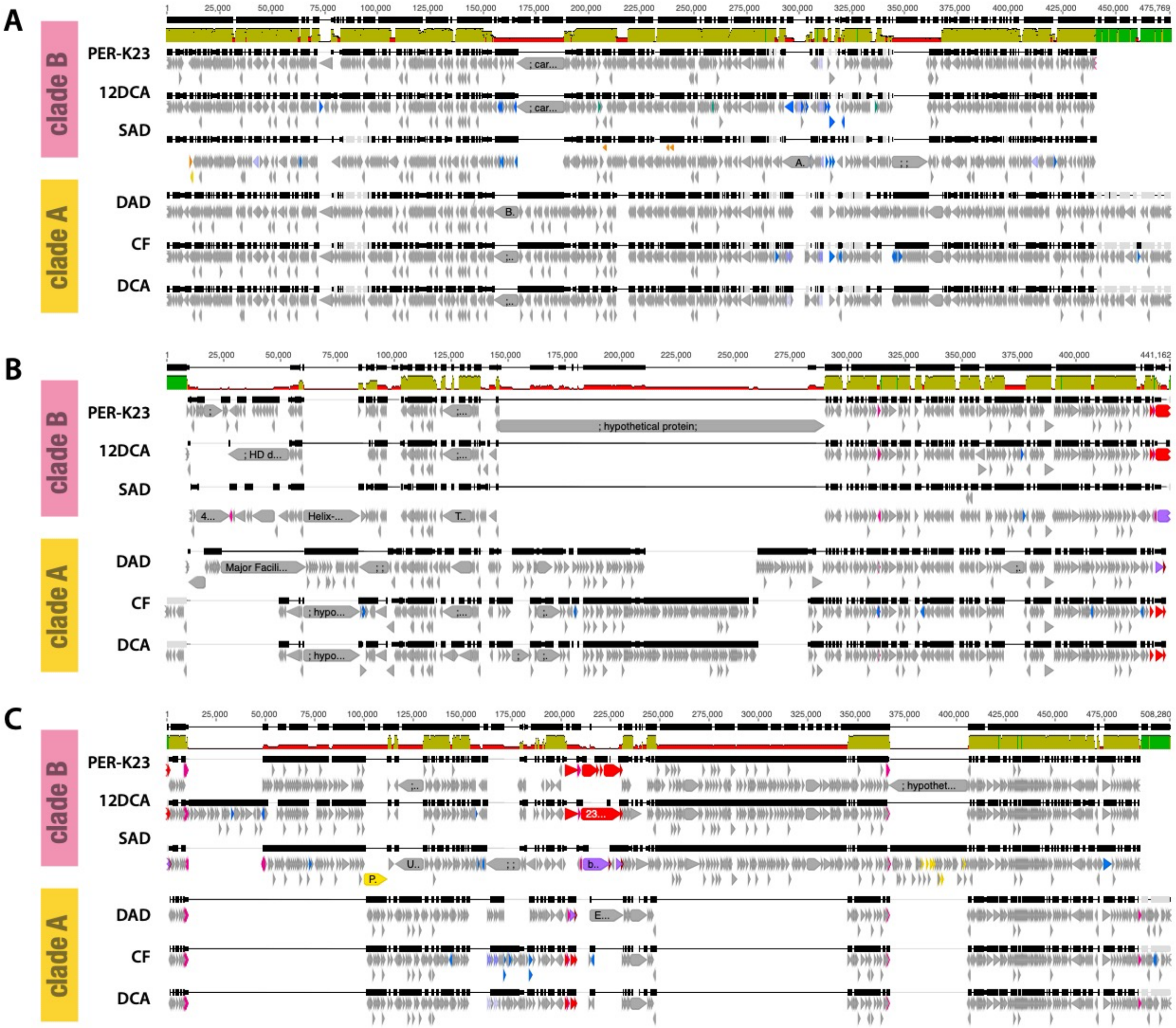

Figure S6. A closer inspection of three locally colinear blocks (LCBs) in the genome-scale alignment of six circularized *Dehalobacter* genomes. Each panel shows a different LCB to demonstrate differences between clades, even in syntenic regions. Length of each LCB is shown at the top of each panel in bp, above the consensus alignment graph, representing the relative similarity at each position.

Figure S7 shows the synteny between each RDase cluster in each complete *Dehalobacter* genome, without extraction and concatenation of *rdhA* sequences, to show that few genes are intersperses between RDase cassettes. We also orient cluster 2 relative to the OG97-*mec* cassette neighborhood (described in our related work (10), and shown in Figure S8), which is close enough to visualize in *Dehalobacter* strains CF and DCA, whereas strains SAD and DAD have ~250 kb of intervening DNA. This also marks the location of the "integration hotspot" discussed in Text S4.

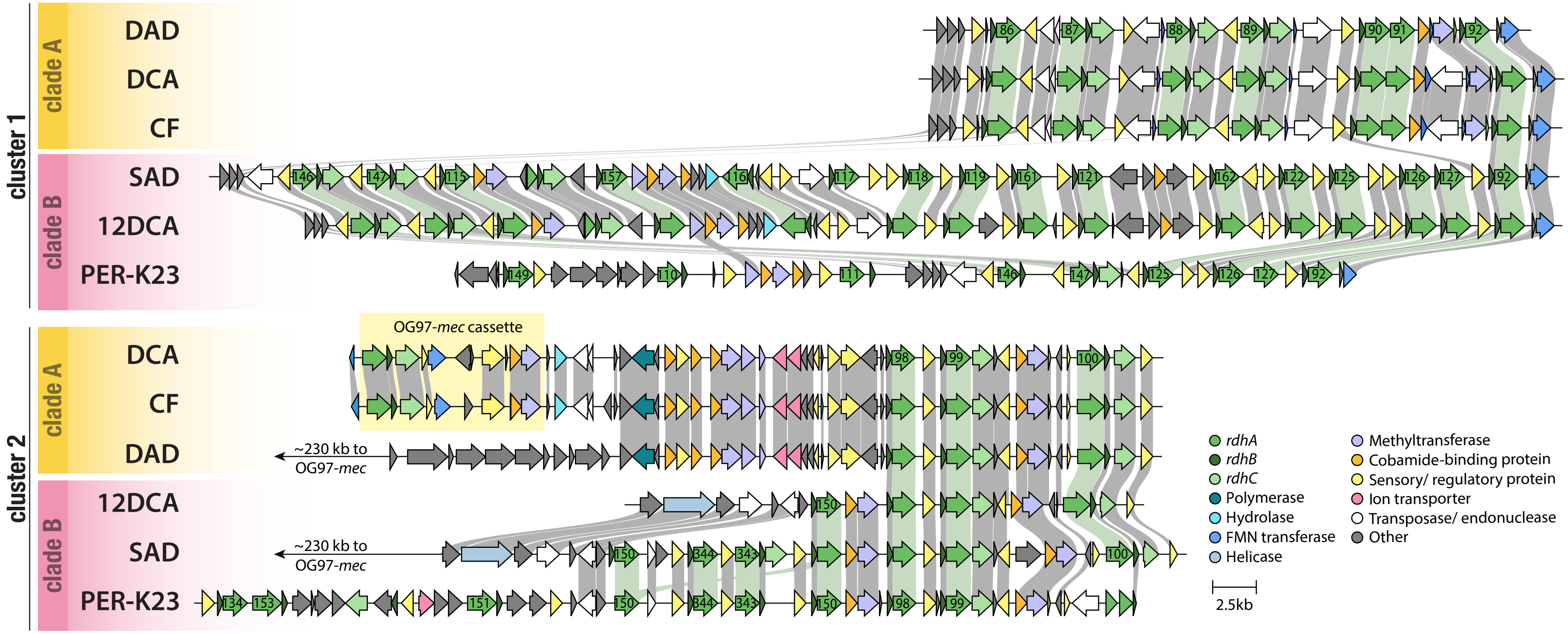

Figure S7. Alignment of genes in RDase clusters from closed *Dehalobacter* genomes. Homologous genes with >90% identity are connected in grey, and RdhA genes in the same OG are connected in green. Cluster 2 is marked in relation to the *mec* cassette in relevant strains. Each clade of *Dehalobacter* is demarcated by colour.

Figure S8 also shows the synteny between the OG97-mec neighbourhood in all *Dehalobacter* strains found to encode it. Strains CF and DCA have lost *mecDEFGHIJ,* possibly due to lack of selective pressure to keep it during their enrichment on 1,1,1-TCA and 1,1-DCA, and no strains share the region downstream of the terminal recombinase shown in the alignment. This may be due to the flexible nature of this "hotspot" region of the genomes detected in the pangenome. Strain XH111TCA also lacks homology upstream of the reverse transcriptase at the 5' end of the gene neighborhood, which may be explained similarly. Closed genomes of strains 8M and XH111TCA would aid in a full understanding of acquisition of this region by diverse *Dehalobacter* strains in Clades A and B.

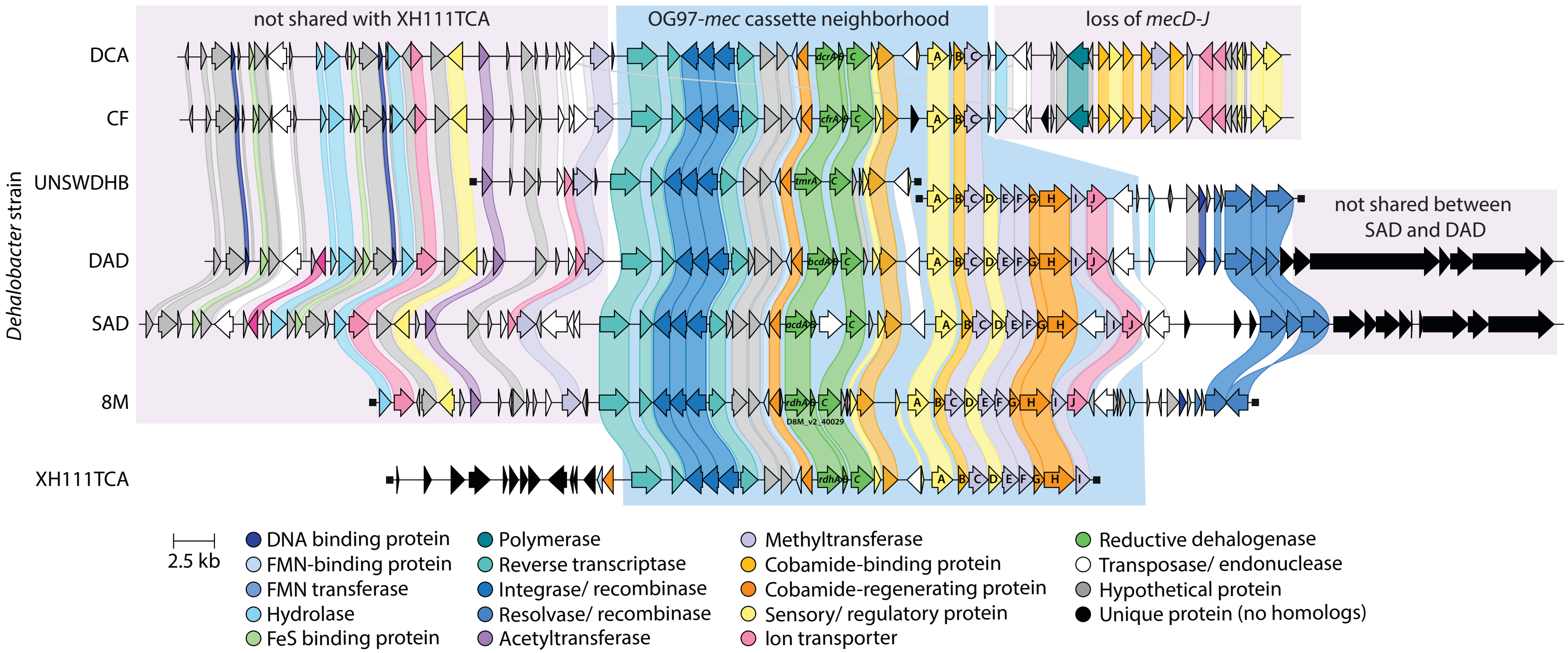

Figure S8. Alignment of the gene neighborhoods containing the OG97 RDase and *mec* cassette from *Dehalobacter* genomes. Homologous genes are linked and color-coded by function. Genes with no local homologs are black. Ends of a contig are capped with black squares.

An RDase phylogeny including select OGs allows closer visualization, so clade-specific relationships can be seen within each OG (Figure S9). One sample OG was selected from RDase cluster 1 (OG92), within which the sequences cluster in a clade-specific manner, suggesting evolution with speciation. Examples of HGT-acquired OGs include OG96 (previously suggested to arise via HGT), and OG100. Some strains encode two OG96 sequences, which the phylogeny suggests was due to HGT across clades, or from a different lineage unrepresented in this tree rather than a duplication ancestral to speciation. The sporadic nature of OG100 clustering also suggests HGT. Examples of OG differentiation due to speciation (OG161/120) and duplication (OG117/118) are also visualized. Though this tree is "rooted" by NpRdhA (because of its distinct host lineage: *Nitratireductor pacificus*), due to the nature of HGT, this may not be accurate rooting to describe the evolutionary "age" of RDase OGs with respect to each other (see unrooted trees in Figure S9B vs. S9C, for example).

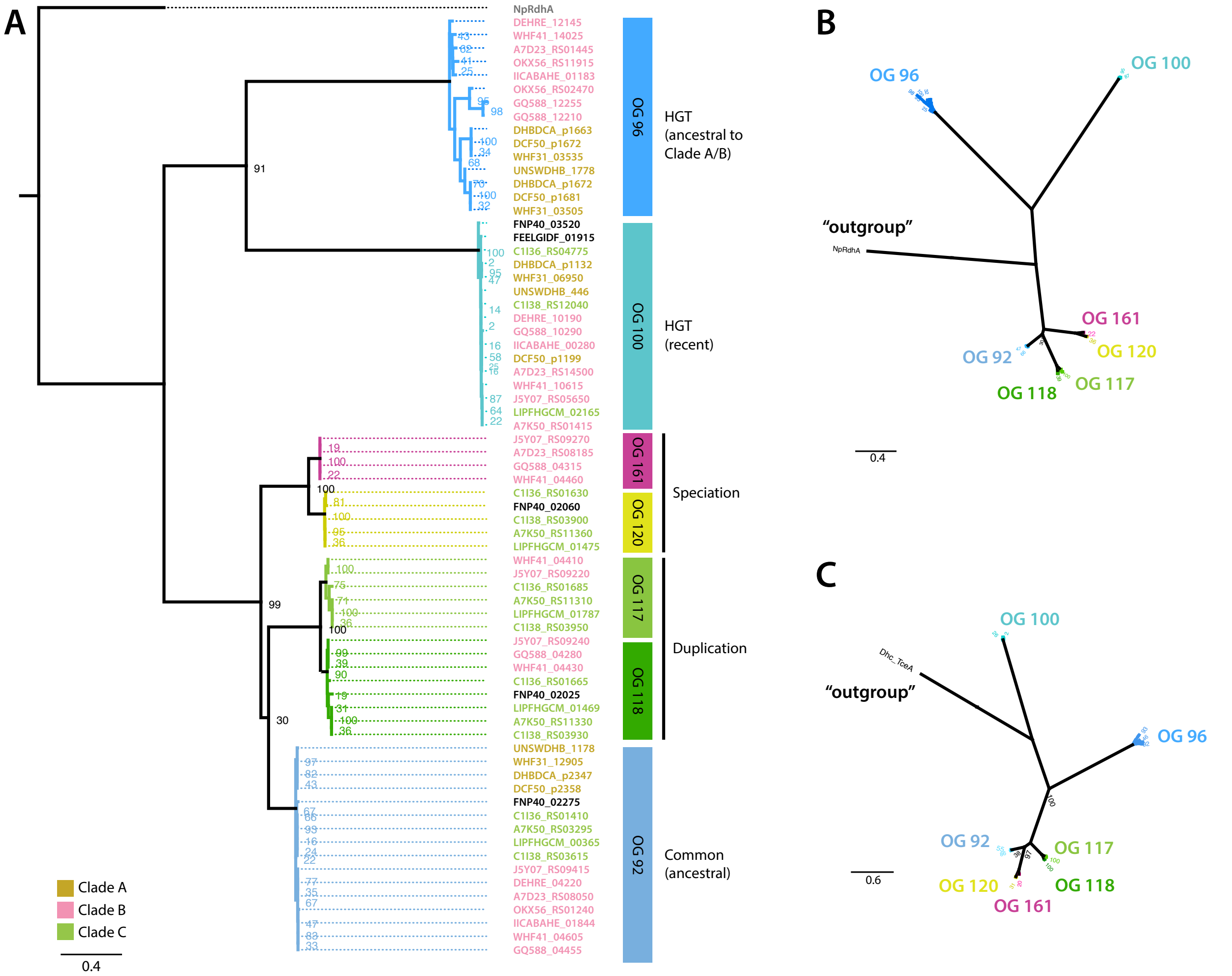

Figure S9. Phylogeny of reductive dehalogenases in select Ortholog Groups (OGs). Best scoring maximum likelihood tree displayed. Scale shows number of substitutions per site. The RDase accessions are coloured by clade, branches are coloured by OG. An RDase from *Nitratireductor pacificus* (NpRdhA, WP_008597722.1) used as an "outgroup" to root the tree in panel A. Trees in panels B and C are unrooted, comparing NpRdhA and TceA from *Dehalococcoides* (AAW39060) as outgroups, respectively.

TEXT S4. Experimental detection of MGE and strain-specific PCR

Here we show the experimental design (Figure S10A-S10B) for PCR amplification and sequencing of integration sites of the predicted MGE. This MGE was predicted in both its circular form and integrated into each *Dehalobacter* genome by PacBio HiFi long read mapping. The integrated and circularized forms of the MGE were both detected via site-specific PCR and sanger sequencing in SC05-UT and DCME (Figure S10C), which were confirmed by sanger sequencing.

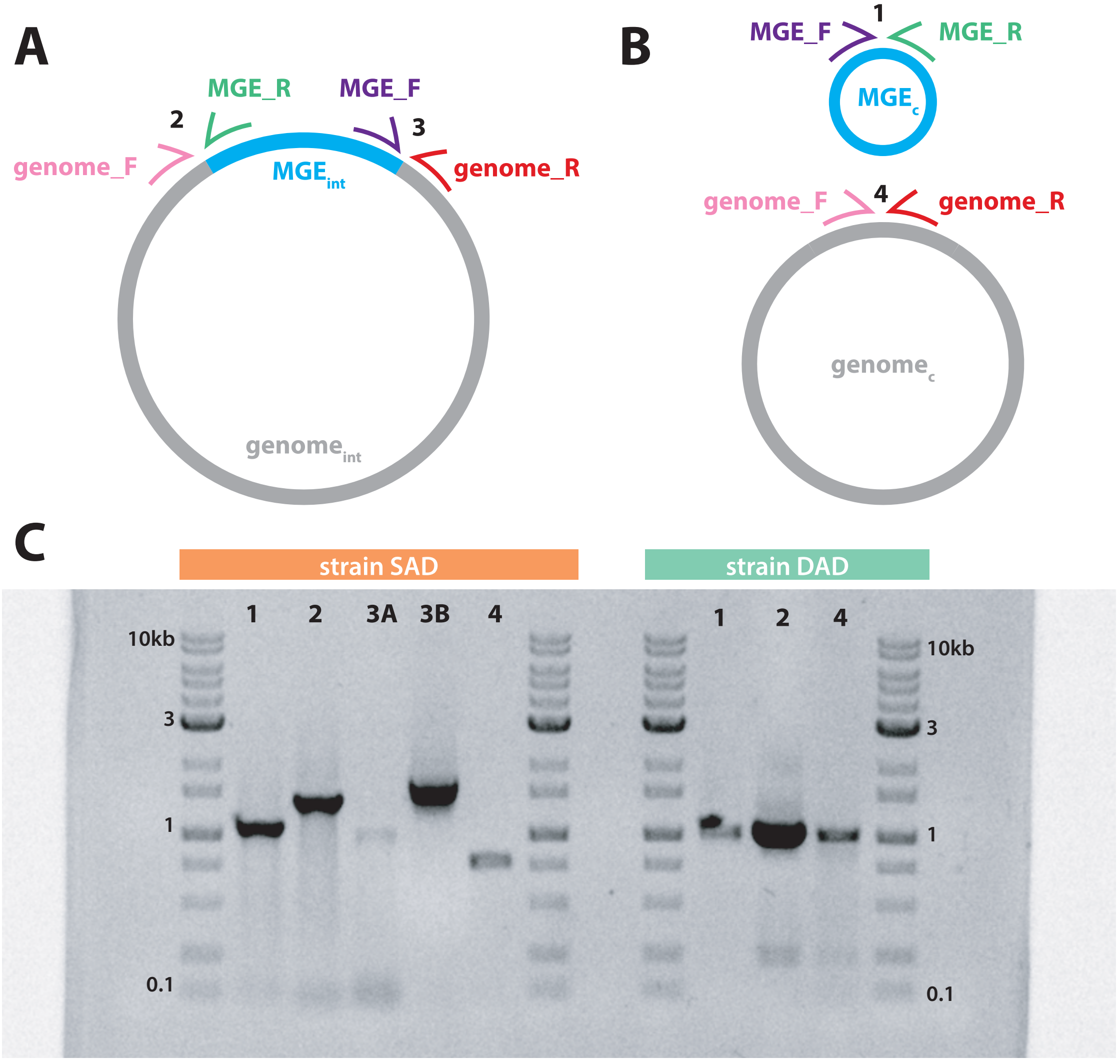

Figure S10. Targeted amplification of circularized and integrated MGEs in SAD and DAD genomes and primer design schematic to amplify key regions when the mobile genetic element is **A)** integrated into the genome, and **B)** circularized/ excised from the genome. Each PCR target region is numbered, 800-1300 bp is between each primer pair. **C)** Gel of amplified targets in strains SAD and DAD. 1) MGE, circular 2) Integrated (upstream) 3) Integrated (downstream, variation A: extra segment, variation B: no extra segment) 4) Genome, gap closed. Ladder: 1kb Plus DNA ladder (FroggaBio, Vaughan, ON).

Table S3. Primer sequences for targeted amplification of circularized and integrated IMEs and genomes, as well as *flgC* strain-specific qPCR (std: qPCR standard).

Genome	Name	Target(s)	Length (bp)	Sequence
DAD	DAD_genome_R	2, 4	23	TTATCCTCCCCGTCAAATTCCTG
	DAD_genome_F	1, 4	23	GCTGTATGGACTGGTACTGGATT
	DAD_MGE_F	2, 3	23	TGCAGGCAAAAGGTTCTTCAAAA
	DAD_MGE_R	1, 3	23	ACATCTCCTGTCTTCTGTGCTTT
	DAD_flgC_S279F	flgC_std	21	AATTGGCCGACAGAATCGAAA
	DAD_flgC_S677R	flgC_std	20	CGTCCGGATTATTCGGATCG
	DAD_flgC_37F	flgC	23	GGTCTTACCGCCCAAAAACTTAG
	DAD_flgC_183R	flgC	23	TTCAGATGGGCGAGAAGAAAAGA
SAD	SAD_genome_R_B	2, 4	20	GCATGTTCAGACGAGACTGC
	SAD_genome_R_A	2, 4	23	TTGATTTTGCGACTGAAGACGAG
	SAD_genome_F_1	1	23	AATGCATATTGATCCCTCGCTCT
	SAD_genome_F_2	4	23	GGCCTTGGCGGATAGTTAATTTC
	SAD_MGE_F	2, 3	23	TTGAGGTCTGGGAAATACTGTGG
	SAD_MGE_R	1, 3	23	GCAGGTATTCCGCAGTTAACAAA
	SAD_flgC_S279F	flgC_std	21	AACTCGCGGATAGAGTTGTAA
	SAD_flgC_S677R	flgC_std	20	CATCCGGGTTATCCGGATCA
	SAD_flgC_33F	flgC	22	GTCGGGTCTCACTGCTCAGAAG
	SAD_flgC_188R	flgC	21	TCCTTCTCCGATGGGCGAGA

TEXT S5. *Dehalobacter* mobilome

This section shows the results from a number of mobilome-specific predictive tools performed on the *Dehalobacter* strain SAD and DAD genomes, as well as proMGE (11) predicted recombinases in the nested-recombinase island "hotspot" of deposited *Dehalobacter* strains (Table S4). *Dehalobacter* strains SAD and DAD were searched for recombinases (Table S5), conjugative elements (Table S6), and prophages (Table S7).

Table S4. Detected recombinases in the nested genomic island of the *Dehalobacter* strains determined by proMGE (11).

***Dehalobacter* strain**	**Transposon type**	**Mobility island type**	**Conjugative element type**	**Phage type**	**Total**
CF	9			1	10
DCA	13		1		14
PER-K23	12	1			13

Table S5A. Detected recombinases in the *Dehalobacter* strain DAD MGE and their classifications, as determined by proMGE (11). MGE modules are coloured to match locally colinear blocks in Figure 7.

**MGE module**	**Gene accession (WHF31_)**	**Recombinase hit**	**Major category**	**Minor category**	**Score**	**E value**	**Length**
	06740	ser_lsr	Phage, CE		341.6	3.0E-104	1,620
	06735	ser_lsr	Phage, CE		391.0	3.2E-119	1,716
	06730	ser_lsr	Phage, CE		389.6	8.8E-119	1,668
	05975	ser_lsr	Phage, CE		348.9	1.8E-106	1,581
	05970	ser_lsr	Phage, CE		377.6	3.8E-115	1,728
	05965	ser_lsr	Phage, CE		404.0	3.9E-123	1,656
	05945	rve	IS_Tn, Phage		91.4	1.7E-28	819
	05885	DDE_Tnp_1_4	IS_Tn		180.7	1.1E-55	1,272
	05570	ser_lsr	Phage, CE		264.8	5.5E-81	762
	05560	ser_lsr	Phage, CE		386.2	9.5E-118	1,599
	05520	DDE_5	IS_Tn		35.6	2.3E-11	1,248
	05460	Transposase_20	IS_Tn		57.1	6.4E-18	864
	05395	RitA	IS_Tn		352.8	1.0E-107	1,236
	05390	RitB	IS_Tn		352.1	9.4E-108	987
	05385	RitC	IS_Tn		441.6	5.7E-135	1,014
	05360	DEDD_Tnp_IS110	IS_Tn	IS	66.7	7.8E-21	351
	05265	DDE_Tnp_1	IS_Tn		35.2	3.7E-11	1,134
	05225	DEDD_Tnp_IS110	IS_Tn	IS	47.6	6.0E-15	849
	05225	Transposase_20	IS_Tn	IS	86.2	5.1E-27	849
	05205	DEDD_Tnp_IS110	IS_Tn	IS	80.0	6.4E-25	1,242
	05205	Transposase_20	IS_Tn	IS	85.4	9.6E-27	1,242

Table S5B. Detected recombinases in the *Dehalobacter* strain SAD MGE region and their classifications, as determined by proMGE (11). MGE modules are coloured to match locally colinear blocks in Figure 7.

MGE module	Gene accession (WHF41_)	Recombinase hit	Major category	Minor category	Score	E value	Length
	10775	DDE_Tnp_1	IS_Tn	IS5H	90	6.30E-28	990
	10785	DEDD_Tnp_IS110	IS_Tn	IS	135.9	3.80E-42	1,290
	10785	Transposase_20	IS_Tn	IS	83.1	4.80E-26	1,290
	10800	ser_lsr	Phage, CE		309.6	1.60E-94	1,248
	10835	ser_lsr	Phage, CE		340	9.10E-104	1,620
	10840	ser_lsr	Phage, CE		388.4	2.00E-118	1,716
	10845	ser_lsr	Phage, CE		386.7	6.50E-118	1,674
	11175	DDE_5	IS_Tn		56.9	7.40E-18	1,443
	11675	rve	IS_Tn, Phage		75.7	1.20E-23	1,377
	11700	ser_lsr	Phage, CE		471.3	1.60E-143	1,563
	11710	ser_lsr	Phage, CE		228.8	4.60E-70	1,152
	11995	ser_lsr	Phage, CE		435.1	1.40E-132	978
	12000	ser_lsr	Phage, CE		393.8	4.60E-120	978
	12040	DDE_5	IS_Tn		35.6	2.30E-11	1,248
	12055	DDE_5	IS_Tn		63.4	7.90E-20	1,443
	12105	Transposase_20	IS_Tn		57.1	6.40E-18	864
	12135	DDE_5	IS_Tn		63.4	7.90E-20	1,443
	12175	RitA	IS_Tn	phage	352.8	1.00E-107	1,236
	12180	RitB	IS_Tn	phage	352.1	9.40E-108	987
	12185	RitC	IS_Tn	phage	441.6	5.70E-135	1,014
	12210	DDE_Tnp_IS66	IS_Tn	IS	307.5	3.50E-94	1,602
	12230	DEDD_Tnp_IS110	IS_Tn	IS	66.7	7.80E-21	351
	12325	DDE_Tnp_1	IS_Tn		35.2	3.70E-11	1,134
	12370	DEDD_Tnp_IS110	IS_Tn	IS	47.6	6.00E-15	849
	12370	Transposase_20	IS_Tn	IS	86.2	5.10E-27	849
	12385	DEDD_Tnp_IS110	IS_Tn	IS	80	6.40E-25	1,242
	12385	Transposase_20	IS_Tn	IS	85.4	9.60E-27	1,242
	NA	ser_lsr	Phage, CE		408.2	2.00E-124	1,650
	NA	ser_lsr	Phage, CE		362.6	1.30E-110	1,632
	NA	DDE_5	IS_Tn		66.8	7.10E-21	1,443

Table S6A. Conjugative and secretion systems detected in the genomes of *Dehalobacter* strains A) DAD and B) SAD and their classifications, including related appendages, as determined by CONJscan and TXSScan (12–14).

Sys ID	System type	Hit ID	Gene	Sys wholeness	Sys score	Hit status	Hit E value
1	Conjugation-T4SS_FATA	WHF31_00535	T4SS_virb4	0.233	4.6	mandatory	3.40E-89
		WHF31_00535	T4SS_virb4	1	2.2	mandatory	3.40E-89
		WHF31_00540	FATA_prgIb	0.233	4.6	accessory	1.40E-11
		WHF31_00560	FATA_prgHa	0.233	4.6	accessory	9.20E-22
		WHF31_00570	FATA_prgF	0.233	4.6	accessory	2.20E-06
		WHF31_00575	T4SS_t4cp2	0.233	4.6	mandatory	2.50E-39
		WHF31_00575	T4SS_t4cp2	1	2.2	mandatory	2.50E-39
		WHF31_00590	FATA_cd411	0.233	4.6	accessory	7.40E-06
		WHF31_00595	T4SS_MOBP1	0.233	4.6	mandatory	1.10E-28
		WHF31_00595	T4SS_MOBP1	1	2.2	accessory	1.10E-28
		WHF31_00815	T4SS_MOBP1	0.233	4.6	mandatory	6.30E-31
		WHF31_00815	T4SS_MOBP1	1	2.2	accessory	6.30E-31
		WHF31_00835	T4SS_t4cp2	0.233	4.6	mandatory	1.40E-38
		WHF31_00835	T4SS_t4cp2	1	2.2	mandatory	1.40E-38
		WHF31_00840	FATA_prgF	0.233	4.6	accessory	6.60E-06
		WHF31_00845	FATA_prgHa	0.233	4.6	accessory	1.80E-20
		WHF31_00860	FATA_prgIb	0.233	4.6	accessory	6.90E-11
		WHF31_00865	T4SS_virb4	0.233	4.6	mandatory	1.60E-92
		WHF31_00865	T4SS_virb4	1	2.2	mandatory	1.60E-92
2	Flagellum	WHF31_04115	Flg_flgC	1	11	mandatory	1.70E-45
		WHF31_04120	Flg_fliE	1	11	mandatory	8.10E-23
		WHF31_04125	Flg_sctJ_FLG	1	11	mandatory	1.40E-114
		WHF31_04140	Flg_sctN_FLG	1	11	mandatory	3.90E-193
		WHF31_04160	Flg_sctQ_FLG	1	11	mandatory	6.10E-08
		WHF31_04165	Flg_sctQ_FLG	1	11	mandatory	6.90E-29
		WHF31_04180	Flg_sctR_FLG	1	11	mandatory	2.50E-96
		WHF31_04185	Flg_sctS_FLG	1	11	mandatory	1.80E-32
		WHF31_04190	Flg_sctT_FLG	1	11	mandatory	2.40E-49
		WHF31_04195	Flg_sctU_FLG	1	11	mandatory	1.10E-119
		WHF31_04200	Flg_sctV_FLG	1	11	mandatory	4.70E-277
		WHF31_04260	Flg_flgB	1	11	mandatory	7.00E-29
3	Tad	WHF31_04950	Tad_tadV	0.5	5	mandatory	1.10E-19
		WHF31_04955	Tad_rcpC	0.5	5	accessory	5.20E-15
		WHF31_04965	Tad_tadA	0.5	5	mandatory	6.80E-35
		WHF31_04970	Tad_tadB	0.5	5	mandatory	1.10E-05
		WHF31_04975	Tad_tadC	0.5	5	mandatory	1.80E-08
		WHF31_10655	Tad_tadE	0.5	5	accessory	3.00E-14
		WHF31_10660	Tad_tadE	0.5	5	accessory	1.60E-14
4	T4aP	WHF31_10345	T4aP_pilO	0.529	6.2	accessory	7.20E-06
		WHF31_10355	T4aP_pilA	0.529	6.2	mandatory	9.40E-18
		WHF31_10360	T4aP_pilC	0.529	6.2	mandatory	2.10E-66
		WHF31_10365	T4aP_pilB	0.529	6.2	mandatory	6.00E-164
		WHF31_10375	T4aP_pilE	0.529	6.2	accessory	7.70E-10
		WHF31_10380	T4aP_pilV	0.529	6.2	accessory	6.40E-11
		WHF31_10400	T4aP_fimT	0.529	6.2	accessory	2.90E-13
		WHF31_10420	T4aP_pilO	0.529	6.2	accessory	8.50E-10
		WHF31_10430	T4aP_pilM	0.529	6.2	accessory	2.60E-14
		WHF31_03865	T4aP_pilD	0.529	6.2	mandatory	4.70E-60
5	Tad	WHF31_10910	Tad_flp	0.667	6.7	mandatory	2.70E-11
		WHF31_10915	Tad_tadE	0.667	6.7	accessory	9.30E-31
		WHF31_10920	Tad_tadG	0.667	6.7	neutral	9.60E-19
		WHF31_10925	Tad_rcpC	0.667	6.7	accessory	2.70E-23
		WHF31_10930	Tad_tadZ	0.667	6.7	mandatory	1.30E-29
		WHF31_10935	Tad_tadA	0.667	6.7	mandatory	1.20E-121
		WHF31_10940	Tad_tadB	0.667	6.7	mandatory	2.10E-41
		WHF31_10945	Tad_tadC	0.667	6.7	mandatory	6.10E-38
		WHF31_04875	Tad_tadV	0.667	6.7	mandatory	4.70E-17
6	T4aP	WHF31_10990	T4aP_pilD	0.588	7.5	mandatory	2.30E-38
		WHF31_10995	T4aP_pilB	0.588	7.5	mandatory	1.50E-174
		WHF31_11000	T4aP_pilT	0.588	7.5	mandatory	1.50E-144
		WHF31_11015	T4aP_pilA	0.588	7.5	mandatory	3.50E-18
		WHF31_11030	T4aP_pilW	0.588	7.5	accessory	1.80E-12
		WHF31_11040	T4aP_pilX	0.588	7.5	accessory	1.60E-08
		WHF31_11045	T4aP_pilO	0.588	7.5	accessory	2.50E-13
		WHF31_11050	T4aP_pilN	0.588	7.5	accessory	2.50E-08
		WHF31_11055	T4aP_pilM	0.588	7.5	accessory	2.20E-25
		WHF31_11060	T4aP_pilC	0.588	7.5	mandatory	1.20E-113

Table 6B. *Continued.*

Sys ID	System type	Hit ID	Gene	Sys wholeness	Sys score	Hit status	Hit E value
7	Conjugation-T4SS_FATA	WHF41_00405	T4SS_virb4	1	2.2	mandatory	1.30E-89
		WHF41_00445	T4SS_t4cp2	1	2.2	mandatory	3.50E-39
		WHF41_00465	T4SS_MOBP1	1	2.2	accessory	2.30E-28
		WHF41_00405	T4SS_virb4	0.233	4.6	mandatory	1.30E-89
		WHF41_00410	FATA_prgIb	0.233	4.6	accessory	2.30E-11
		WHF41_00430	FATA_prgHa	0.233	4.6	accessory	7.80E-22
		WHF41_00440	FATA_prgF	0.233	4.6	accessory	2.20E-06
		WHF41_00445	T4SS_t4cp2	0.233	4.6	mandatory	3.50E-39
		WHF41_00460	FATA_cd411	0.233	4.6	accessory	1.00E-05
		WHF41_00465	T4SS_MOBP1	0.233	4.6	mandatory	2.30E-28
8	Conjugation-T4SS_FATA	WHF41_02020	T4SS_MOBP1	1	2.2	accessory	7.20E-29
		WHF41_02035	T4SS_t4cp2	1	2.2	mandatory	2.40E-37
		WHF41_02085	T4SS_virb4	1	2.2	mandatory	5.00E-93
		WHF41_02020	T4SS_MOBP1	0.233	4.6	mandatory	7.20E-29
		WHF41_02025	FATA_cd411	0.233	4.6	accessory	2.80E-06
		WHF41_02035	T4SS_t4cp2	0.233	4.6	mandatory	2.40E-37
		WHF41_02040	FATA_prgF	0.233	4.6	accessory	0.00038
		WHF41_02065	FATA_prgHa	0.233	4.6	accessory	3.70E-22
		WHF41_02080	FATA_prgIb	0.233	4.6	accessory	4.60E-12
		WHF41_02085	T4SS_virb4	0.233	4.6	mandatory	5.00E-93
9	Tad	WHF41_02115	Tad_tadB	0.5	4.5	mandatory	5.20E-11
		WHF41_02120	Tad_tadC	0.5	4.5	mandatory	7.00E-07
		WHF41_02135	Tad_tadE	0.5	4.5	accessory	0.00032
		WHF41_02145	Tad_tadV	0.5	4.5	mandatory	2.00E-15
		WHF41_12755	Tad_tadC	0.5	4.5	mandatory	2.80E-08
		WHF41_12765	Tad_tadA	0.5	4.5	mandatory	6.80E-42
		WHF41_12775	Tad_rcpC	0.5	4.5	accessory	1.20E-13
10	T4aP	WHF41_06305	T4aP_pilT	0.588	7	mandatory	4.70E-144
		WHF41_06310	T4aP_pilB	0.588	7	mandatory	2.70E-175
		WHF41_06315	T4aP_pilD	0.588	7	mandatory	1.50E-38
		WHF41_06865	T4aP_pilM	0.588	7	accessory	1.50E-14
		WHF41_06875	T4aP_pilO	0.588	7	accessory	1.80E-09
		WHF41_06895	T4aP_fimT	0.588	7	accessory	2.80E-13
		WHF41_06915	T4aP_pilV	0.588	7	accessory	4.30E-10
		WHF41_06920	T4aP_pilE	0.588	7	accessory	9.90E-10
		WHF41_06930	T4aP_pilB	0.588	7	mandatory	2.00E-164
		WHF41_06935	T4aP_pilC	0.588	7	mandatory	9.70E-67
		WHF41_06940	T4aP_pilA	0.588	7	mandatory	8.50E-18
		WHF41_06950	T4aP_pilO	0.588	7	accessory	4.00E-08
11	Tad	WHF41_06360	Tad_tadC	0.667	6.7	mandatory	1.40E-38
		WHF41_06365	Tad_tadB	0.667	6.7	mandatory	1.40E-41
		WHF41_06370	Tad_tadA	0.667	6.7	mandatory	7.50E-122
		WHF41_06375	Tad_tadZ	0.667	6.7	mandatory	3.90E-32
		WHF41_06380	Tad_rcpC	0.667	6.7	accessory	6.70E-23
		WHF41_06385	Tad_tadG	0.667	6.7	neutral	3.20E-18
		WHF41_06390	Tad_tadE	0.667	6.7	accessory	1.00E-30
		WHF41_06395	Tad_flp	0.667	6.7	mandatory	4.10E-11
		WHF41_12720	Tad_tadV	0.667	6.7	mandatory	4.80E-17
12	Tad	WHF41_06635	Tad_tadE	0.5	5	accessory	3.00E-14
		WHF41_06640	Tad_tadE	0.5	5	accessory	1.60E-13
		WHF41_12620	Tad_tadC	0.5	5	mandatory	1.90E-08
		WHF41_12625	Tad_tadB	0.5	5	mandatory	1.10E-05
		WHF41_12630	Tad_tadA	0.5	5	mandatory	6.90E-35
		WHF41_12640	Tad_rcpC	0.5	5	accessory	5.20E-15
		WHF41_12645	Tad_tadV	0.5	5	mandatory	1.10E-19
13	Flagellum	WHF41_13310	Flg_flgB	1	11	mandatory	1.50E-28
		WHF41_13370	Flg_sctV_FLG	1	11	mandatory	1.10E-278
		WHF41_13375	Flg_sctU_FLG	1	11	mandatory	4.20E-118
		WHF41_13380	Flg_sctT_FLG	1	11	mandatory	2.40E-50
		WHF41_13385	Flg_sctS_FLG	1	11	mandatory	6.90E-33
		WHF41_13390	Flg_sctR_FLG	1	11	mandatory	1.20E-96
		WHF41_13405	Flg_sctQ_FLG	1	11	mandatory	7.00E-29
		WHF41_13410	Flg_sctQ_FLG	1	11	mandatory	3.20E-09
		WHF41_13430	Flg_sctN_FLG	1	11	mandatory	7.30E-193
		WHF41_13445	Flg_sctJ_FLG	1	11	mandatory	1.20E-114
		WHF41_13450	Flg_fliE	1	11	mandatory	2.10E-21
		WHF41_13455	Flg_flgC	1	11	mandatory	8.00E-47
14	MOB	WHF41_15055	T4SS_t4cp2	0.667	1.2	accessory	1.00E-10
		WHF41_15065	T4SS_MOBC	0.667	1.2	mandatory	1.70E-09

Table S7. Putative prophage-derived regions in the *Dehalobacter* strain SAD and DAD genomes, as determined by phaster (15).

Region	Region length (Kb)	Region Locus (Kb)	Completeness (Score)	Total proteins	Phage hits	Hypothetical proteins	ATT site	GC (%)
DAD-1	20.2	656-677	incomplete(10)	10	6	4	present	41.0
DAD-2	17.4	1068-1085	incomplete(30)	6	6	0	present	40.4
DAD-3	34.1	1099-1134	incomplete(50)	11	8	3	present	47.8
DAD-4	45.7	2489-2534	questionable(78)	65	51	14	absent	59.8
DAD-5	24.8	3101-3125	incomplete(10)	15	11	4	present	41.6
DAD-6	16.7	3284-3301	incomplete(30)	23	12	11	present	46.1
SAD-1	40.5	488-529	incomplete(60)	53	38	15	absent	45.0
SAD-2	14.5	2334-2348	incomplete(30)	21	15	6	absent	40.7
SAD-3	28.2	2351-2379	incomplete(30)	36	25	11	absent	43.2
SAD-4	31.3	2419-2450	incomplete(40)	10	8	2	present	40.8
SAD-5	26.5	2882-2909	incomplete(50)	14	7	7	present	45.0
SAD-6	13.4	3024-3037	incomplete(30)	14	11	3	absent	46.3

TEXT S6. A survey of the genomic island modules

Strains DCA and 12DCA share a colinear block downstream of *rumA* (orange, Figure 7). The genome of strain SAD also optionally harbours this motif in only one of its closed forms (described in Supplemental Text 1). This region encodes a serine recombinase system followed by a regulatory module, DNA methylases, and a possible Type 2/4 secretion system, though many genes are only annotated as hypothetical proteins (Figure S11). These features suggest this region to be a separate 87.6 kb mobile element that may be transmissible separately from the rest of the MGE. Though the genomes of strains CF and DCA are almost identical, strain CF lacks this region, indicating a somewhat recent insertion or excision (25).

Within the blue and pink LCBs in Figure 6, there is a cassette of DNA-transfer related genes that are homologous with the orange LCB (Figure S11). In the blue LCB, upstream of the transfer cassette are metal-binding and redox proteins which are likely cargo brought in via HGT with integration of this ICE. This motif has no leading recombinase but may have originally been an independent ICE that has since lost functionality with the loss of its recombinase genes. This can also be seen in the last 60 kb of the pink LCB, which has genetic cargo carrying redox-related proteins and acyltransferases, but no nearby recombinase (Figure S11).

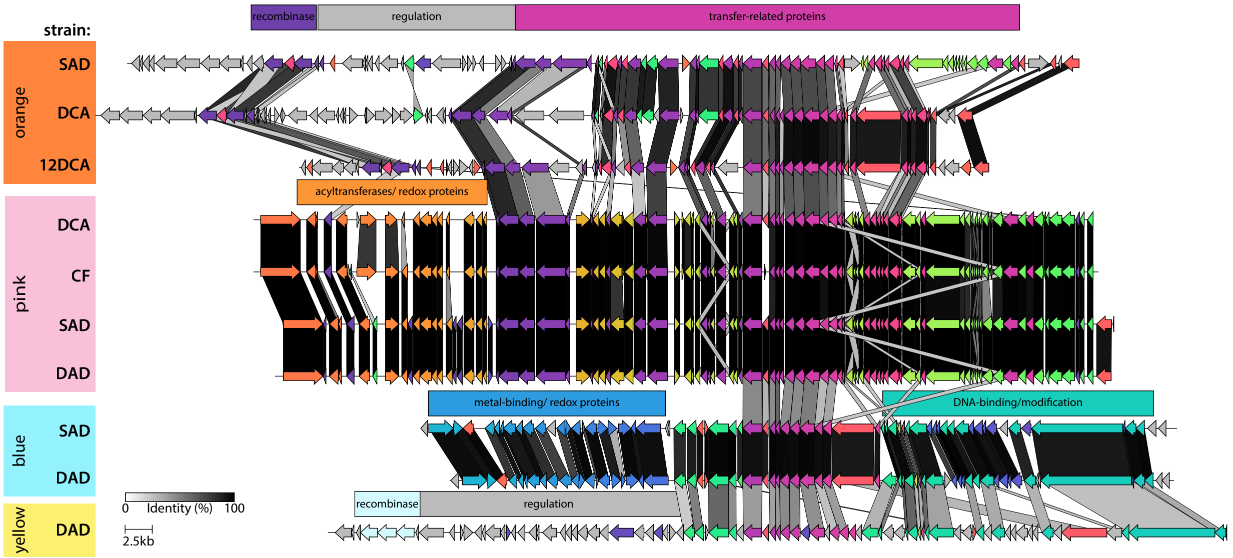

Figure S11. Gene structure of LCB motifs encoding putative DNA transfer systems in HGT region extracted from all closed *Dehalobacter* genomes. Coding regions are coloured by homology, and each cluster is labelled with a putative function.

One additional copy of this transfer cassette is found in the strain DAD genomic island, in the region highlighted in yellow in Figure 6. This region has similar structure to the orange motif, with a leading recombinase followed by regulatory and transfer proteins but ends with a cluster of DNA binding and

modification proteins (Figure S11). This also implies previous integration of this region as an independent ICE. Notably, this region is not present in the SAD genomic island and may represent a more recent integration into the island. In the SAD spot, a region highlighted in green replaces this yellow region (Figure 6). This region harbours a series of 8-10 prophage genes, as detected by Phaster (15), and exhibits a lower GC content on each end than the surrounding areas (30% GC). While this is no longer a complete phage, "recent" phage integration may also constitute the difference between these otherwise highly similar spots in strains SAD and DAD.

The pink region is shared by strains CF, DCA, SAD, and DAD, and encodes a number of ICE-specific genes, such as anti-restriction proteins, a Type IV secretion system, *mobL* relaxase, integrases and reverse transcriptases, in addition to our cargo of interest: the *mec* cassette and OG97 RDase (Figure S8). Strains CF and DCA do not contain a full *mec* cassette; they only possess the genes for *mecABC*. Interestingly, their active RDases also belong to OG97 and are present on this ICE upstream of *mecA*. The region surrounding the SAD and DAD *mec* cassettes and OG97 RDase are peppered with transposases and endonucleases, which may have played a role in the genes acquisition or deletion (10). There are also some predicted phage genes surrounding the RDase (Table S7), so the route of transmission remains unclear.

SI References

1. Bulka O, Edwards EA. 2024. Two distinct *Dehalobacter* metagenome-assembled genomes from anaerobic chloroform and dichloromethane degrading consortia. Microbiol Resour Announc forthcoming.

2. Bulka O, Edwards EA. 2024. Metagenomic sequences from anaerobic chloroform and dichloromethane degrading microbial communities. Microbiol Resour Announc 13:e00391-24. DOI:10.1128/mra.00391-24.

3. Antipov D, Korobeynikov A, McLean JS, Pevzner PA. 2016. HybridSPAdes: An algorithm for hybrid assembly of short and long reads. Bioinformatics 32:1009–1015. DOI:10.1093/bioinformatics/btv688.

4. Tang S, Gong Y, Edwards EA. 2012. Semi-Automatic In Silico Gap Closure Enabled De Novo Assembly of Two *Dehalobacter* Genomes from Metagenomic Data. PLoS One 7:e52038. DOI:10.1371/journal.pone.0052038.

5. Deshpande NP, Wong YK, Manefield M, Wilkins MR, Lee M. 2013. Genome Sequence of *Dehalobacter* UNSWDHB, a Chloroform-Dechlorinating Bacterium. Genome Announc 1:720–733. DOI:10.1128/genomea.00720-13.

6. Kruse T, Maillard J, Goodwin L, Woyke T, Teshima H, Bruce D, Detter C, Tapia R, Han C, Huntemann M, Wei CL, Han J, Chen A, Kyrpides N, Szeto E, Markowitz V, Ivanova N, Pagani I, Pati A, Pitluck S, Nolan M, Holliger C, Smidt H. 2013. Complete genome sequence of *Dehalobacter restrictus* PER-K23. Stand Genomic Sci 8:375–388. DOI:10.4056/sigs.3787426.

7. Molenda O, Puentes Jácome LA, Cao X, Nesbø CL, Tang S, Morson N, Patron J, Lomheim L, Wishart DS, Edwards EA. 2020. Insights into origins and function of the unexplored majority of the reductive dehalogenase gene family as a result of genome assembly and ortholog group classification. Environ Sci Process Impacts 22:663–678. DOI:10.1039/c9em00605b.

8. Alfán-Guzmán R, Ertan H, Manefield M, Lee M. 2017. Genome sequence of *Dehalobacter* sp. strain TeCB1, able to respire chlorinated benzenes. Genome Announc 5:e01681-16. DOI:10.1128/genomea.01681-16.

9. Puentes Jacome LA. 2019. Anaerobic Biodegradation of Chlorinated Benzenes and Hexachlorocyclohexane by Mixed Microbial Cultures Derived from Contaminated Field Sites. University of Toronto. DOI:https://hdl.handle.net/1807/97603.

10. Bulka O, Picott K, Mahadevan R, Edwards EA. 2024. From *mec* cassette to *rdhA*: a key *Dehalobacter* genomic neighborhood in a chloroform and dichloromethane–transforming microbial consortium. Appl Environ Microbiol 90:1–24. DOI:10.1128/aem.00732-24.

11. Khedkar S, Smyshlyaev G, Letunic I, Maistrenko OM, Coelho LP, Orakov A, Forslund SK, Hildebrand F, Luetge M, Schmidt TSB, Barabas O, Bork P. 2022. Landscape of mobile genetic elements and their antibiotic resistance cargo in prokaryotic genomes. Nucleic Acids Res 50:3155–3168. DOI:10.1093/nar/gkac163.

12. Cury J, Abby SS, Doppelt-Azeroual O, Néron B, Rocha EPC. 2020. Identifying Conjugative Plasmids and Integrative Conjugative Elements with CONJscan, p. 265–283. *In* Methods in Molecular Biology. DOI:10.1007/978-1-4939-9877-7_19.

13. Abby SS, Cury J, Guglielmini J, Néron B, Touchon M, Rocha EPC. 2016. Identification of protein secretion systems in bacterial genomes. Sci Rep 6:23080. DOI:10.1038/srep23080.

14. Guglielmini J, Eron BN, Abby SS, Garcilí An-Barcia MP, De La Cruz F, Rocha EPC. 2014. Key components of the eight classes of type IV secretion systems involved in bacterial conjugation or protein secretion. Nucleic Acids Res 42:5715–5727. DOI:10.1093/nar/gku194.

15. Arndt D, Grant JR, Marcu A, Sajed T, Pon A, Liang Y, Wishart DS. 2016. PHASTER: a better, faster version of the PHAST phage search tool. Nucleic Acids Res 44:W16–W21. DOI:10.1093/nar/gkw387.
